# Semirandom DNA adducts regulate a filamentous defense-associated reverse transcriptase

**DOI:** 10.1038/s41594-026-01813-8

**Published:** 2026-06-10

**Authors:** Nolan Neville, Nicole V. Johnson, Edwin E. Escobar, Chang-Hwa Chiang, Albana Nreca, Sean R. Johnson, Nan Dai, Andy Hanneman, Ivan R. Corrêa, Jason S. McLellan, Robert J. Trachman

**Affiliations:** 1 New England Biolabs Inc., Ipswich, MA USA; 2 Department of Molecular Biosciences, The University of Texas at Austin, Austin, TX USA; 3 École Supérieure de Biotechnologie de Strasbourg, Strasbourg, France

**Keywords:** Cryoelectron microscopy, Phage biology

## Abstract

Retrons and several defense-associated reverse transcriptases (DRTs) synthesize non-genomic DNA for bacteriophage immunity. In some instances, this non-genomic DNA is of undefined, semirandom sequence. How undefined DNA sequences impart antiphage defense is not known. Herewe report the cryo-EM structure and functional characterization of the DRT1 antiphage defense system. We show that DRT1 performs template-free, protein-primed DNA synthesis to generate semirandom DNA adducts. DNA synthesis activates the nitrilase domain of DRT1, while DNA adducts drive the assembly of quiescent DRT1 filaments. Filamentous DRT1 is composed of domain-swapped C termini that are entwined, forming pseudoknots between tetrameric stacks. This configuration occludes conserved active-site residues, resulting in a dormant state. Bacteriophage escape mutants identify a T4 single-stranded DNA helicase required for DRT1 activity. Functionally, DRT1 resembles a minimal retron where a single gene produces a reverse transcriptase, effector and non-genomic antitoxin DNA.

## Abstract (web summary)

Neville et al. show that DRT1 synthesizes semirandom DNA adducts to stabilize a filamentous state. C-terminal pseudoknots form along the axis of the filament. The pseudoknot conformation inhibits a nitrilase domain, preventing abortive infection.

## Main

Bacteria have evolved diverse pathways to contest bacteriophage. One broadly implemented defense strategy is to degrade foreign nucleic acids^1–3^. In contrast with this tactic, defense-associated reverse transcriptases (DRTs) synthesize DNA to impart immunity. DRTs are part of the so-called unknown group (UG) of reverse transcriptases (RTs), which are closely related to the abortive infection (Abi) RTs. Collectively, these are referred to as UG/Abi RT systems. Groundbreaking work by several research teams^4–6^ has identified dozens of UG/Abi RT families, which are categorized into three major classes based on functional gene annotation or domain association^6^. Class 1 UG/Abi RTs homo-oligomerize to perform template-free, protein-primed DNA synthesis^7,8^. This process results in a homo-oligomeric protein core with DNA adducts of seemingly random sequences. The function of undefined single-stranded DNA (ssDNA) in antiphage defense has yet to be determined.

Like retrons^9–11^, class 2 UG/Abi RT operons encode an RT along with a noncoding RNA and sometimes an additional protein. Despite this similarity, the two characterized class 2 DRTs, DRT2 and DRT9, are mechanistically distinct and unparalleled in nature. Retron RTs produce multicopy single-stranded DNA (msDNA) as an autoregulatory antitoxin that stabilizes filaments and senses phage infection^12–14^, and class 2 DRTs produce DNA templates encoding toxic products^15–18^.

Despite a wide distribution across diverse bacterial taxa, class 3 UG/Abi RTs are the least characterized. Class 3 UG/Abi RTs are defined by multidomain proteins bearing a RT domain and either a nitrilase or phosphohydrolase domain^6^. These systems are intriguing, as neither nitrilases nor phosphohydrolases are common to antiphage defense systems^5,19^. These attributes suggest that class 3 UG/Abi RTs function through an unprecedented mechanism.

In this Article, using cryo-EM and functional assays, we investigate the mechanism of the class 3 DRT1 antiphage defense system. We demonstrate that class 3 DRTs perform protein-primed, template-free DNA synthesis to impart immunity to bacteriophage. DRT1-DNA adducts serve to activate the nitrilase domain by inducing a tetrameric state while simultaneously imparting a dormant filamentous state. Bacteriophage escape mutants identify the T4 5′→3′ single-stranded DNA helicase, Dda, as part of the activating network of DRT1. Collectively, our results reveal how DRT1-DNA adducts promote a pre-active state primed for inducing programmed cell death upon infection.

## A diverse class of DRTs perform template-free protein-primed DNA synthesis

**Fig. 1:**
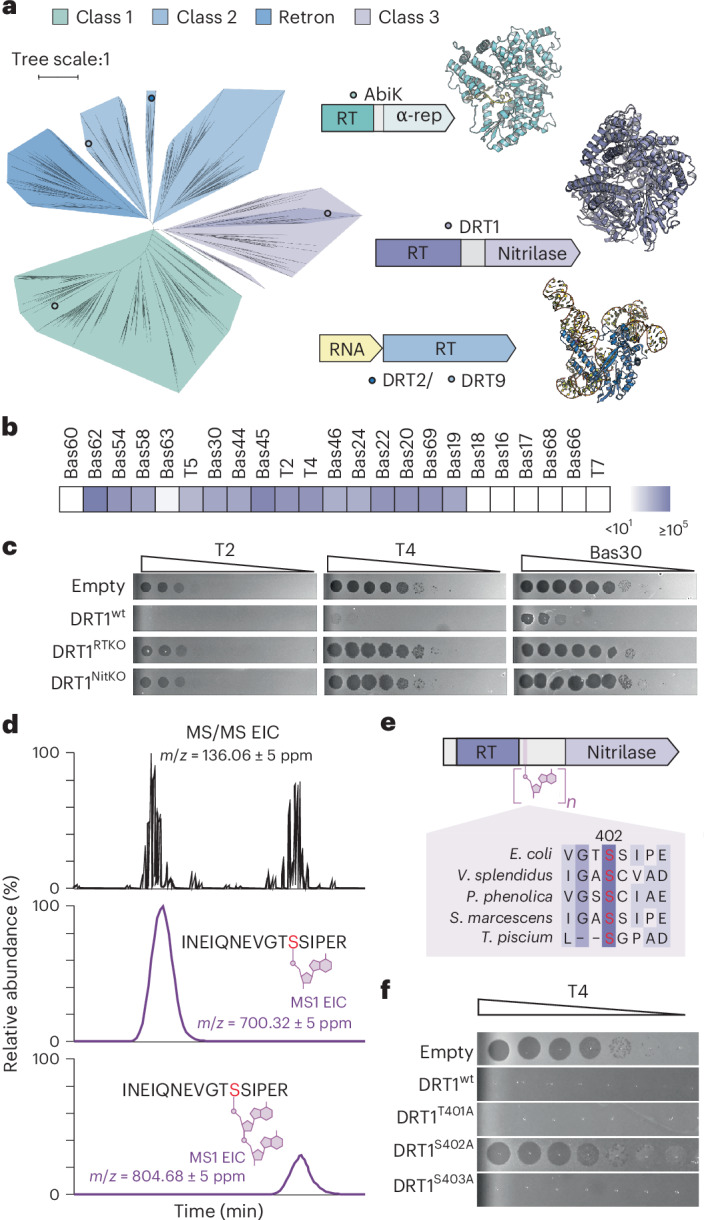
Antiphage defense via protein-primed DNA synthesis. **a**, Phylogenetic analysis of 31 UG/Abi RT families and retrons^4^. Clades are colored by UG/Abi RT class, as described by Mestre and others^6^. The cartoon representations of enzymes and enzyme complexes represent published structures of AbiK^7^ and DRT2^15^ or the AlphaFold2^20^ model for DRT1. **b**, Heatmap representing plaquing of tenfold serial dilutions of bacteriophage on lawns of *E. coli* MG1655. Darker shades indicate greater protection against bacteriophage. Data are the average of *n* = 3 independent experiments. **c**, Plaquing of tenfold bacteriophage serial dilutions on lawns of *E. coli* MG1655 with control (empty vector), DRT1^wt^ and active-site mutants. Representative images of three biological replicates are shown. **d**, LC-MS traces of trypsin-digested DRT1, with detection of the adenine diagnostic fragment. EIC, extracted ion chromatogram. **e**, Cartoon representation of DRT1 identifying the relative location of the serine priming site. The limited sequence alignment window focuses on conserved serine 402. **f**, Plaquing of tenfold bacteriophage serial dilutions on lawns of *E. coli* MG1655 with control (empty vector) and expressed DRT1 and DRT1 priming-site mutants. Representative images of three biological replicates are shown. Source data

DRTs within the UG/Abi RTs are grouped into three classes, defined by their association with conserved domains^6^. Phylogenetic analysis of 31 previously annotated UG/Abi RT families and retrons revealed that class 3 DRTs reside in a distinct clade (Fig. 1a), suggesting a unique structure and mechanism. Indeed, the predicted structure of the class 3 DRT1 by AlphaFold2^20^ (pLDDT = 86.2) is distinct from those of class 1 and 2 DRTs, with the RT and nitrilase domains forming a compact globular shape (Fig. 1a). These data support a structural role for these domains but do not address any functional enzymatic requirement for either the RT or nitrilase domain in antiphage defense.

**Extended Data Fig. 1:**
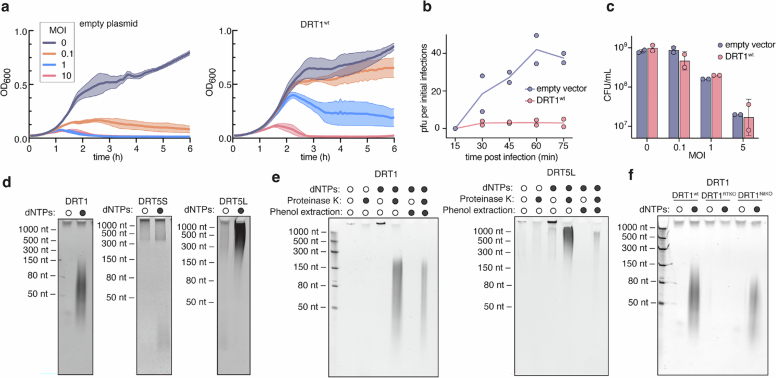
DRT1 antiphage activity and assay of DNA synthesis by purified DRT1, DRT5S, and DRT5L. **a)** Growth curve of cells expressing empty plasmid or DRT1^wt^ +/− T4 phage at the indicated multiplicity of infection (MOI). Data are shown as mean ± s.d. for n = 3 independent biological replicates. **b)** Burst size of T4 phage infecting cells with or without DRT1^wt^. Data are shown as mean ± s.d. for n = 2 independent biological replicates per condition. **c)** Cell survival assay of bacteria following 15 min of T4 phage infection. Each sample shows n = 2 biological replicates, with bars denoting the mean and s.d. **d)** dNTP polymerization assay of purified DRT1, DRT5S, and DRT5L. Samples were treated with proteinase K, then resolved on 15% TBE-urea gel. A representative gel of n = 2 independent experiments is shown for each sample. **e)** Covalent attachment of DNA to DRT1 and DRT5 as demonstrated by the necessity of proteinase K digestion to release DNA into the soluble phase during phenol-chloroform extraction. A representative gel of n = 2 independent experiments is shown for each sample. **f)** dNTP polymerization assay of purified WT (DRT1^wt^), RT-inactive (DRT1^RTKO^), and nitrilase-inactive (DRT1^NitKO^) protein. A representative gel of n = 2 independent experiments is shown. Source data

We performed spot titer assays to test the function of DRT1 and its individual domains using a bacteriophage library composed of T-phages and a subset of the Basel collection^21^. In the surrogate host *Escherichia coli* MG1655, DRT1 provides robust antiphage defense against a diverse subset of representative bacteriophage (Fig. 1b). Mutagenesis of either the DRT1 RT active-site residues D324N/D325N (DRT1^RTKO^) or nitrilase active-site residue C1116A (DRT1^NitKO^) abolished immunity to bacteriophage (Fig. 1c). Consistent with abortive infection, DRT1 only provided immunity at low multiplicities of infection (MOIs) and reduced bacteriophage burst size while maintaining the colony survival rate (Extended Data Fig. 1a–c). Like the RT-toprim fusion of retron-Eco2^22^, enzymatic activity of the RT and effector domains of DRT1 is required to induce abortive infection. Together, these data support a defense mechanism distinct from either class 1 or class 2 DRTs.

To experimentally test the biochemical properties of class 3 DRTs, we expressed and purified DRT1, DRT5S and DRT5L. Reminiscent of the class 1 UG/Abi RTs, class 3 DRTs synthesized DNA upon incubation with dNTPs in the absence of a supplemented RNA template and in the presence of RNase A (Extended Data Fig. 1d). This feature is indicative of protein-primed DNA synthesis, a mechanism observed in bacteriophage polymerases^23,24^ and antiphage defense systems^7,8,18,25^, which results in covalent fusion between protein and DNA. Accordingly, we chose to test whether DNA is covalently bound to DRT1 and DRT5. Post reaction with dNTPs, DNA products were only resolved by electrophoresis for samples digested with proteinase K (Extended Data Fig. 1e). Furthermore, both DRT1^wt^ and DRT1^NitKO^ synthesized DNA (Extended Data Fig. 1f). Additionally, intact mass spectrometry of DRT1^wt^ resolved masses of apo-DRT1 and DRT1•dAMP (Extended Data Fig. 3a,b). These data support that class 3 DRTs produce covalent DNA adducts through template-free, protein-primed, DNA synthesis, independent of nitrilase activity.

Focusing on DRT1, we sought to determine the amino-acid priming site by developing a tandem mass spectrometry (MS/MS) method following a similar approach developed for the detection of *O*-glycosylation in proteins^26^. Following tryptic digestion, a higher-energy collisional dissociation (HCD)-triggered-EThcD MS/MS method was used to observe nucleotide-specific diagnostic fragment ions confirming covalently bound nucleotides. This analysis revealed S402 as the priming site, as evidenced by the detection of four key fragment ions on a single dA-modified peptide. Up to four deoxyadenosine residues were observed on this priming site (Fig. 1d and Extended Data Figs. 3c and 4). S402 is highly conserved within the DRT1 family (Fig. 1e), but not in other class 3 DRTs. To validate the priming site, we mutated S402 and adjacent residues T401 and S403 to alanine. The S402A mutation completely abolished bacteriophage immunity, but the T401A and S403A mutations had no effect (Fig. 1f). The use of a single serine residue for DRT1 priming is a notable distinction from other protein-primed UG/Abi RTs.

## DNA adducts promote a dormant filamentous state of DRT1

**Fig. 2:**
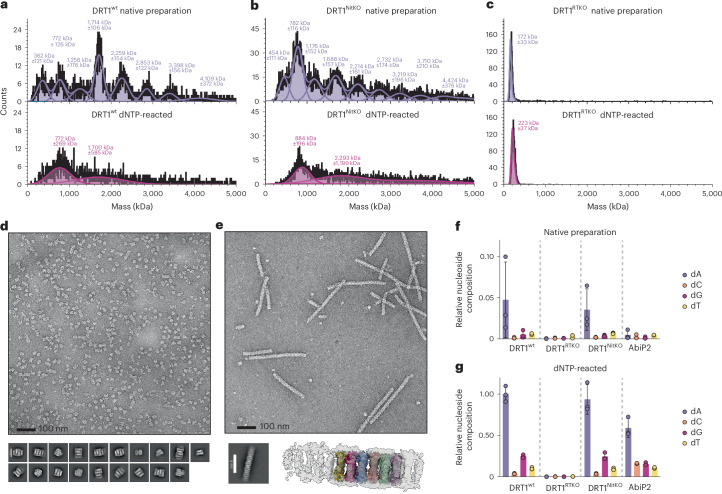
DNA adducts drive filament formation. **a**–**c**, Mass photometry of DRT1^wt^ (**a**), DRT1^NitKO^ (**b**) and DRT1^RTKO^ (**c**) from native preparation (purple) and dNTP-reacted (magenta) samples. Fits to read counts are shown as solid lines, with average and standard error (s.e.) of masses labeled above peaks. **d**, Negative-stain electron micrograph of DRT1 from native preparation. 2D class averages are shown below the micrograph image. **e**, Negative-stain electron micrograph of dNTP-reacted DRT1. 2D class averages of filaments are shown below the micrograph, along with a 3D reconstruction of the filament with AlphaFold2-predicted DRT1 tetramers (colored models) fit to electron density. **f**, Relative nucleoside composition of DRT1^wt^, DRT1 variants and AbiP2 from native preparation. Each sample shows *n* = 3 biological replicates, with data presented as mean and s.d. **g**, Relative nucleoside composition of dNTP-reacted DRT1^wt^, DRT1 variants and AbiP2. Each sample shows *n* = 3 biological replicates, with data presented as mean and s.d. Source data

**Extended Data Fig. 2:**
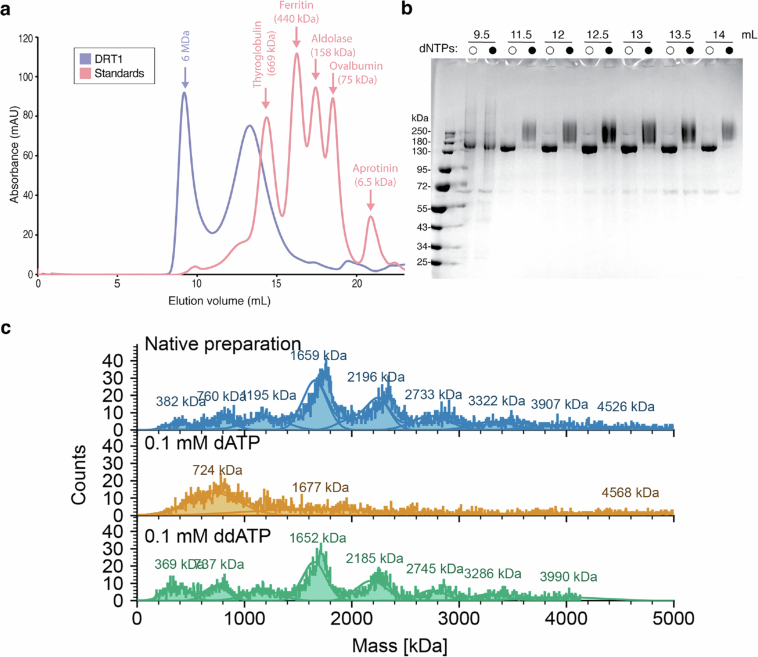
Oligomeric state of DRT1. **a)** Superose 6 10/300 GL size exclusion chromatogram of DRT1^wt^ overlaid with a chromatogram of protein standards and their relative masses. **b)** DRT1^wt^ fractions (elution volume in mL) from **(a)**, either reacted with dNTPs (filled circles) or unreacted (open circles), then resolved by SDS–PAGE (gel of n = 1) **c)** Mass photometry of DRT1^wt^ native preparation, or reacted with dATP or ddATP. Source data

**Extended Data Fig. 3:**
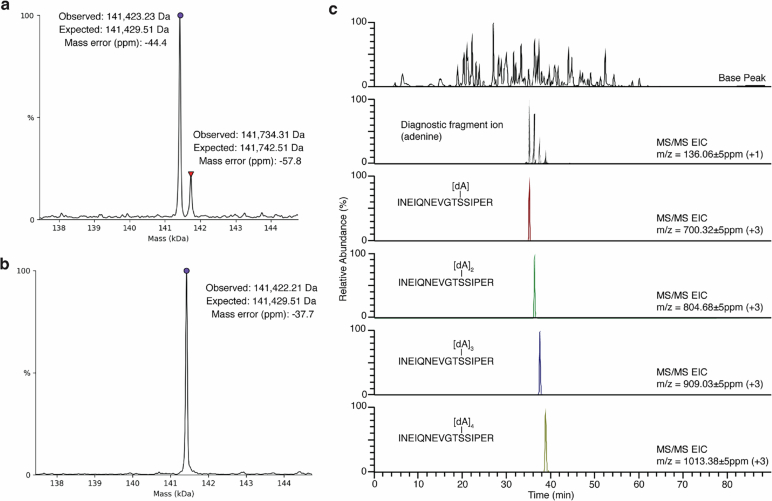
Intact, denatured LC-MS deconvoluted spectra of DRT1. **a)** DRT1^wt^, and **b)** DRT1^RTKO^. DRT1^wt^ shows the addition of mass of approximately 312 Da, consistent with deoxyadenosine monophosphate (313 Da) addition. **c)** Reverse phase base peak traces of DRT1^wt^ tryptic digest with extracted ion chromatogram highlighting deoxyadenosine [dA]-containing peptides. Extracted ion chromatograms of the peptide INEIQNEVGTSSIPER ( + 3 charge state) with 1–4 deoxyadenosines showing sequential elution of the single dA-bound species eluting first, followed by the sequential elution of each +1[dA] species.

**Extended Data Fig. 4:**
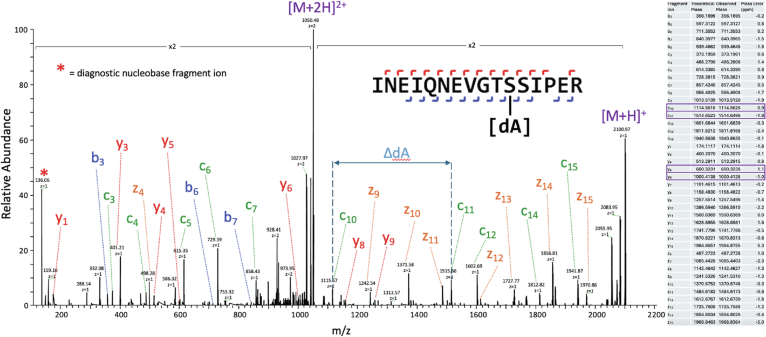
HCD product-ion triggered EThcD mass spectrum of DRT1^wt^ tryptic peptide containing a bound deoxyadenosine. Adduct peptide sequence is (INEIQNEVGTS[dA]SIPER, 2 + , m/z 1050.48). A peptide sequence MS/MS coverage map is shown in the upper right corner of the MS/MS spectrum, and a table of observed fragment ions within ±10ppm mass error of the expected mass. Key nucleotide localizing fragments are outlined in purple.

DRT1 exhibited unusual properties during purification. Size exclusion chromatography (SEC) revealed a broad elution peak corresponding to a mass range of ~440 kDa to ~3 MDa, with a second peak eluting with an estimated mass of ~6 MDa (Extended Data Fig. 2a). Analysis of individual SEC fractions by sodium dodecyl sulfate polyacrylamide gel electrophoresis (SDS–PAGE) under reducing conditions revealed only the presence of DRT1^wt^ (Extended Data Fig. 2b). We performed mass photometry on DRT1^wt^, DRT1^NitKO^ and DRT1^RTKO^ to further understand the mass distribution of DRT1. The mass profile of natively prepared DRT1^wt^ and DRT1^NitKO^ comprised eight or more species with average masses approximating to multiples of tetrameric DRT1 (Fig. 2a,b). Upon reaction with dNTPs, the mass profiles became monodispersed. As confirmed by SDS–PAGE (Extended Data Fig. 2b), these data are consistent with protein-primed DNA synthesis causing shifts to high molecular weight. The DRT1^RTKO^ mutant was monomeric (Fig. 2c), confirming the requirement of DRT1-DNA adducts to form high-order oligomeric states. The reaction of DRT1^wt^ with dideoxy ATP did not alter the oligomer distribution (Extended Data Fig. 2c). Together, these data indicate that DNA adducts are required for filamentation.

**Extended Data Fig. 5:**
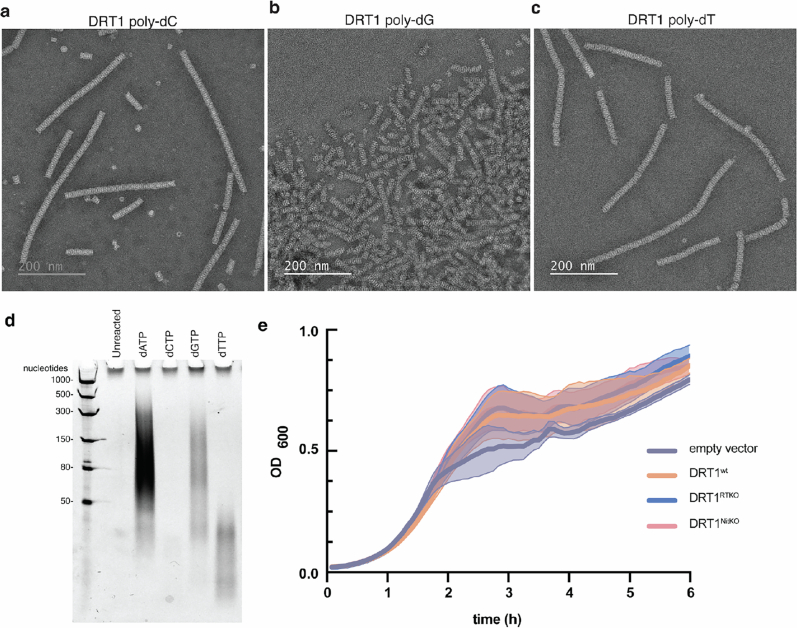
Filament formation, nucleotide preference, and absence of DRT1 toxicity in the absence of phage. **a-c**, Negative-stain EM of DRT1^wt^ reacted with **a**) dCTP, **b**) dGTP, or **c**) dTTP. A representative micrograph of n = 3 samples is shown for each. **d)** dNTP polymerization assay gel of DRT1^wt^ reacted with the indicated individual dNTPs. A representative gel of n = 3 independent experiments is shown. **e)** Growth curve of cells expressing empty plasmid or DRT1 in the absence of phage. Data are shown as mean ± s.d. for n = 3 independent biological replicates. Source data

Next, we performed negative-stain electron microscopy on unreacted and dNTP-reacted DRT1 to understand the organization of DRT1 oligomers and how DNA synthesis alters these assemblies. Electron micrographs of natively prepared DRT1 revealed a heterogenous population of protein assemblies (Fig. 2d). The resulting 19 two-dimensional (2D) population class averages show a distribution of stacked planar assemblies forming truncated filaments tens of nanometers in length. The reaction of DRT1 with dNTPs increased the filament length to >100 nm (Fig. 2e). Consistent with the tetrameric repeats observed by mass photometry, AlphaFold2 predicted tetramers of DRT1 fit to the 3D reconstruction of the reacted sample. Furthermore, the nucleobase composition of the DNA adducts did not impact filament formation (Extended Data Fig. 5a–c), despite DRT1 exhibiting a higher propensity to incorporate dATP in cells and in vitro (Fig. 2f,g and Extended Data Fig. 5d). However, dGTP did promote shorter, aggregated DRT1 filaments. This observation is likely a result of inter-protomer and inter-filament G-quadruplexes^27^. These data demonstrate that DRT1 expressed in *E. coli* contains DNA adducts and forms filamentous structures prior to bacteriophage infection. Given that the expression of DRT1^wt^, or the active-site mutants, does not alter the growth kinetics of *E. coli* (Extended Data Fig. 5e), the filamentous and monomeric forms of DRT1 are not toxic, requiring phage infection to induce cell death.

## Overall structure of DRT1

**Fig. 3:**
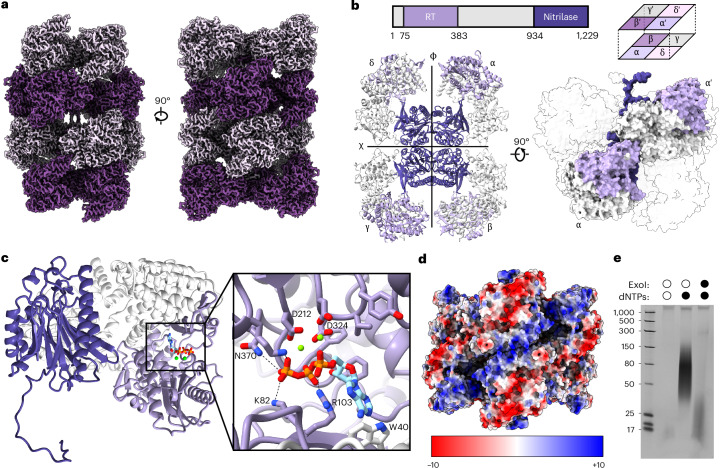
Cryo-EM structure of the DRT1 filament. **a**, Electron density (EM) map of the DRT1 filament. **b**, Arrangement and nomenclature of the DRT1 protomers. Protomers are labeled α−δ in the tetramer model. A surface representation showing inter-protomer interactions between tetramer stacks is shown on the right. **c**, Cartoon representation of the DRT1 monomer colored by domain, with the inset showing the dATP substrate in the RT active site. **d**, The DRT1 octamer shown as a molecular surface, colored by electrostatic potential (−10 to 10 kT e^−1^). **e**, Urea–PAGE of DRT1 DNA adducts from the native preparation, post reaction with dNTPs, and post reaction with dNTPs and exonuclease I digest. A representative gel of *n* = 3 experiments is shown. ExoI, exonuclease I. Source data

**Extended Data Fig. 6:**
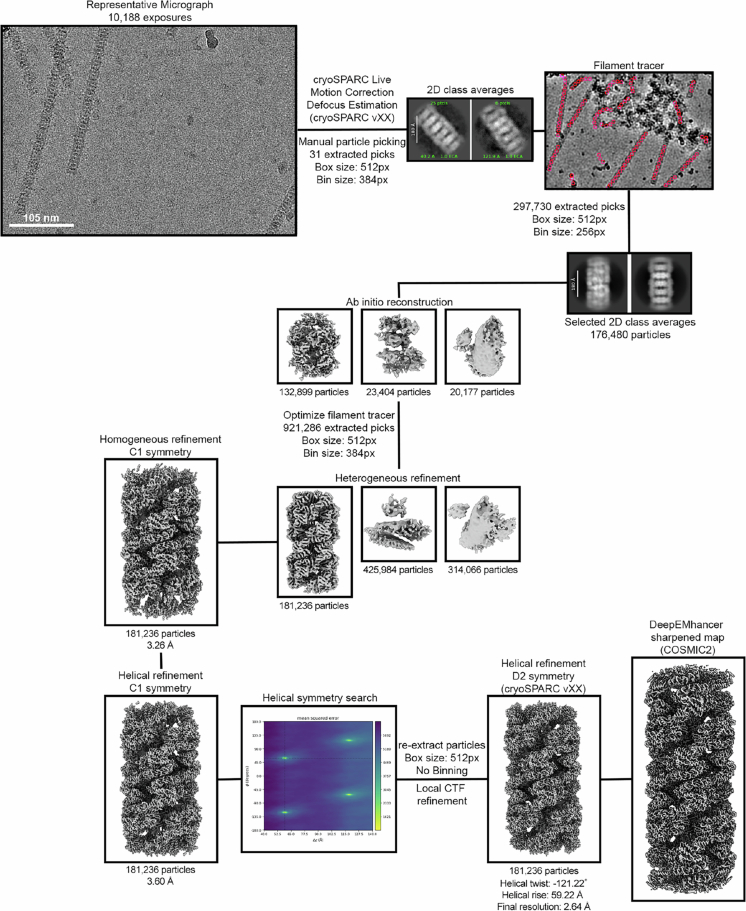
Cryo-EM data processing workflow. Flowchart illustrating the cryo-EM data processing steps used to obtain the structure of the DRT1 filament. Particle counts at each stage are indicated, along with resolutions and symmetry parameters for the 3D reconstructions.

To further investigate the molecular basis for DRT1 filament assembly, we performed single-particle cryo-EM on dNTP-reacted DRT1^wt^. Ab initio reconstruction and heterogeneous refinement of particles extracted from the filaments yielded a high-quality 3D reconstruction revealing a right-handed helical structure composed of stacked DRT1 tetramers. A helical symmetry search performed in cryoSPARC yielded a high-confidence candidate symmetry pair (twist = −121.22°, rise = 59.13 Å) corresponding to a repeating octameric DRT1 unit. These parameters were used in subsequent helical refinement. A final reconstruction of 140,634 particles with additionally imposed *D*2 point group symmetry yielded a 2.6-Å-resolution reconstruction of the DRT1 filament (Fig. 3a, Table 1 and Extended Data Fig. 6). The high-resolution cryo-EM reconstruction allowed for unambiguous model building of the DRT1 octamer. Residues 382–478, including the priming site residue S402, and 837–846 were not resolved in the map.

**Table 1:** Cryo-EM data collection, refinement and validation statistics

	DRT1 + dNTPs (EMD-72883), (PDB 9YFD)
**Data collection and processing**	
Magnification	105,000
Voltage (kV)	300
Electron exposure (e^−^/Å^2^)	55
Defocus range (μm)	0.9–2.4
Pixel size (Å)	0.826
Symmetry imposed	D2
Initial particle images (no.)	1,134,805
Final particle images (no.)	181,263
Map resolution (Å)	2.6
FSC threshold	0.143
Map resolution range (Å)	1.79–28.25
**Refinement**	
Initial model used (PDB code)	AlphaFold 3 model
Model resolution (Å)	2.7
FSC threshold	0.5
Map sharpening *B* factor (Å^2^)	−88.1
Model composition	
Nonhydrogen atoms	73,480
Protein residues	9,000
Ligands	24 (8 ATP, 16 Mg^2+^)
*B* factors (Å^2^)	
Protein	38.6
Ligand	94.0
R.m.s. deviations	
Bond lengths (Å)	0.004
Bond angles (°)	1.03
Validation	
MolProbity score	1.02
Clashscore	1.61
Poor rotamers (%)	0.42
Ramachandran plot	
Favored (%)	97.41
Allowed (%)	2.59
Disallowed (%)	0

The DRT1 tetramer is defined with monomeric units arranged clockwise as α, β, γ and δ with protomer interfaces ϕ and χ (Fig. 3b). Tetramers assemble with the RT and α-helical repeat domains forming the filament periphery and the nitrilase domains forming the core. Intra-tetramer contacts are exclusively mediated by residues from the nitrilase domain. In total, 4,278 Å^2^ of surface area is buried between the nitrilase ϕ (2,614 Å^2^) and χ (1,664 Å^2^) faces. The octameric subunit of DRT1 filaments is composed of tetramers stacked along a central axis. Successive tetramers are flipped by 180° along the plane of the tetramer and rotated by 60.61° (Fig. 3b). This repeating arrangement results in inter-tetramer van der Waals and electrostatic interactions between the RT and the α-helical repeat domain between residues 689–692 and 696–715 to 199–204, 332–336 and 431–437.

**Extended Data Fig. 7:**
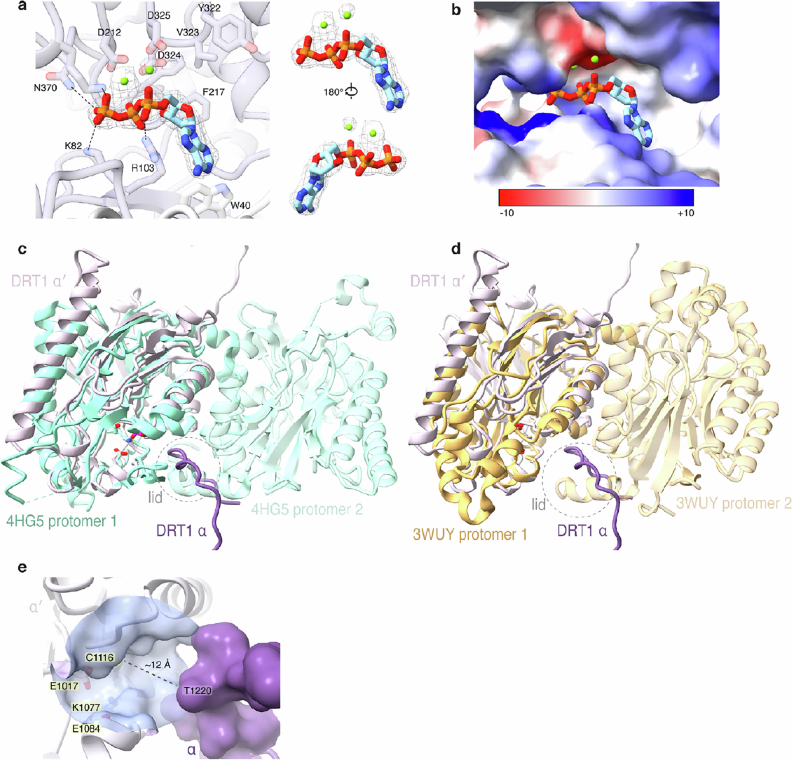
DRT1 RT active site and comparison of the DRT1 nitrilase domain. **a)** DRT1 with dATP substrate in the RT active site. Electron density for the dATP substrate shown as mesh contoured at threshold: 0.169. **b)** Electrostatic surface representation of the DRT1 RT active site pocket. **c)** Superposition of the DRT1 α′ protomer nitrilase domain (residues 934-1229) (pink) with protomer 1 of yeast Nit2 (PDB: 4HG5) (teal) ‘closed lid’ conformation. Oxaloacetate substrate of Nit2 protomer 1 is shown in magenta, and nitrilase catalytic triad residues of both proteins are shown as sticks. **d)** Superposition of the DRT1 α′ protomer nitrilase domain (residues 934-1229) (pink) with protomer 1 of *Syechocystis* sp. nitrilase (PDB: 3WUY) (yellow) ‘open lid’ conformation. Nitrilase catalytic triad residues of both proteins are shown as sticks. **e)** Surface representation of the DRT1 nitrilase active site pocket formed between protomers α′ and α. There is approximately 12 Å between the catalytic C1116 of α′ and T1220 on the α lid.

Despite our determination that ssDNA synthesis is responsible for stabilization of the filamentous structure, we were unable to resolve map density corresponding to ssDNA on the DRT1 surface. The only nucleic acid resolved in our structure is a single deoxyadenosine triphosphate molecule coordinated by two Mg^2+^ ions in the RT active site (Fig. 3c and Extended Data Fig. 7a,b). DNA adduct interactions are probably electrostatic, given the lack of sequence dependence on filamentation^28^. To further investigate how ssDNA may interact with the DRT1 filament, the electrostatic potential was calculated for the DRT1 octamer. The electrostatic surface potential revealed an uninterrupted helical patch of positive potential at the interface between tetramers, with an approximate length of 130 Å per tetramer (Fig. 3d). Natively purified or exonuclease I-treated DRT1 results in DNA adducts of ~17–30 nucleotides (Fig. 3e). These data are consistent with DNA adduct nuclease protection correlating in length with the basic helical patch.

## C-terminal domain swapping

**Fig. 4:**
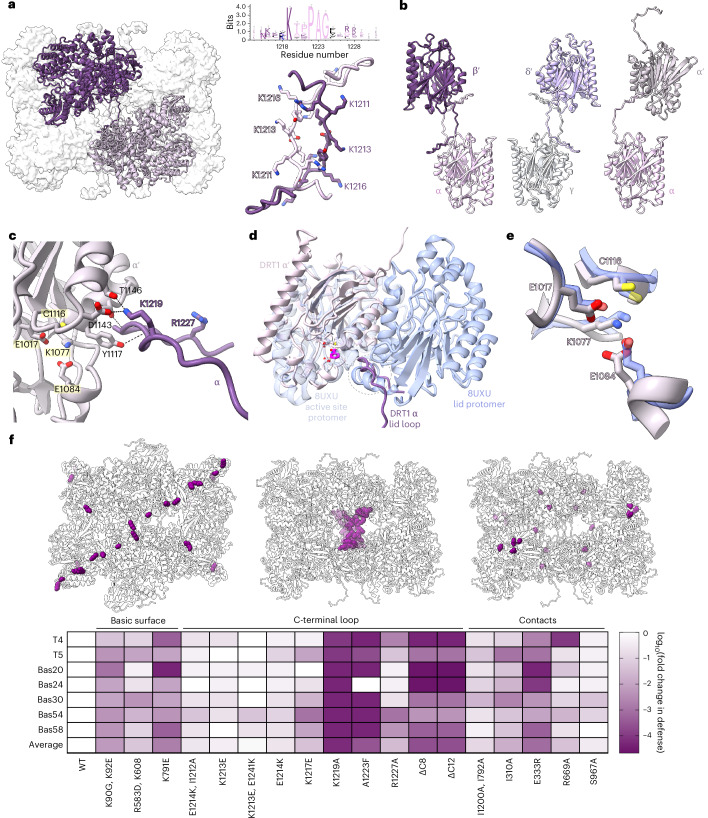
Inter-tetramer interactions are required for DRT1 activity. **a**, The DRT1 octamer, with domain-swapped C termini shown in the inset. The sequence logo of the C-terminal residues is generated from alignment of 174 DRT1 sequences. **b**, Orientation of protomers with domain swapping interactions. A prime symbol (′) indicates a protomer from a different tetramer stack. **c**, Interactions between proximal residues to the nitrilase active site (light pink) and the swapped C terminus of the adjacent protomer (purple). Nitrilase catalytic tetrad residues are labeled in yellow. **d**, DRT1 nitrilase domain (light pink) superimposed with the dimeric unit of closed-lid conformation nitrilase from *Rhodococcus* sp. V51B (PDB 8UXU) (blue) and its benzaldehyde substrate (magenta). The domain-swapped C terminus of a DRT1 protomer is shown in purple, and the nitrilase active-site catalytic tetrad is shown as sticks. The dashed circle shows the lid region. **e**, Superposition of the DRT1 nitrilase active-site catalytic tetrad (light pink) with that of a nitrilase filament from *Rhodococcus* sp. V51B (PDB 8UXU) (blue). **f**, Structure-guided mutations suggested to disrupt DRT1 activity, with mutation locations shown as purple spheres in the structures, and a heatmap of phage plaquing assay relative to DRT1^wt^. Darker shades indicate loss of immunity. The heatmap represents the average of *n* = 3 independent experiments. Source data

In addition to extensive inter-tetramer contacts, the final 19 C-terminal (K1211–H1229) residues extend across the interstitial space of the octamer, resulting in two domain-swapped tetramers per octamer (Fig. 4a). The C termini from protomers on opposite tetramer stacks wrap around each other in an antiparallel orientation to form a pseudoknot (Fig. 4a,b). The inter C-terminal domain contact is stabilized by cation–π interactions between R1210 and Y1215, as well as van der Waals interactions between I1212 and Y1215 (Fig. 4a). This conformation positions C-terminal residues 1218–1221 over the apo-nitrilase binding pocket of the protomer adjacent to the pseudoknot pair (Fig. 4c). This produces a closed-lid conformation similar to those observed between adjacent protomers in bacterial and plant nitrilases (Fig. 4d and Extended Data Fig. 7c,d)^29,30^, with only modest differences in the conformation of the catalytic tetrad (Fig. 4e). The domain-swapped closed-lid conformation of DRT1 results in an occluded substrate binding pocket completed by the C-terminal residues of the adjacent protomer (Extended Data Fig. 7e).

To test the structural and functional significance of DRT1 filament features, we performed a spot titer assay on mutants of DRT1^wt^, evaluating the plaquing efficiency of *E. coli* inoculated with one of seven phages. Single or double amino-acid substitutions were selected to target basic surface residues hypothesized to interact with DNA adducts, residues involved in C-terminal domain swapping, and inter-tetramer contacts (Fig. 4f). The most striking reduction in antiphage defense resulted from substitutions to conserved residues in the C-terminal loop region, with a reduction in plaque formation of up to four orders of magnitude higher than DRT1^wt^. Point substitutions K1219A and A1223F in this region reduced plaquing to levels comparable to C-terminal deletions, ∆1217–1229 (∆C12) and ∆1221–1229 (∆C8), revealing these residues to be essential for DRT1 activity. By contrast, non-conserved residues in the C-terminal loop resulted in ≤10-fold reduction in plaquing. Except for E333R, substitutions targeting tetrameric contacts had a modest impact on plaquing. Maintenance of antiphage defense activity in these mutants is unsurprising because of the substantial amount of buried surface area between DRT1 tetramers. Inter-tetramer contact mutants modestly reduce antiphage phage defense. However, these results do not directly address the structure of DRT1 in the activated state, as these mutations might also impact DNA binding and protection in the dormant state.

## Viral determinants of DRT1 immunity

**Extended Data Fig. 8:**
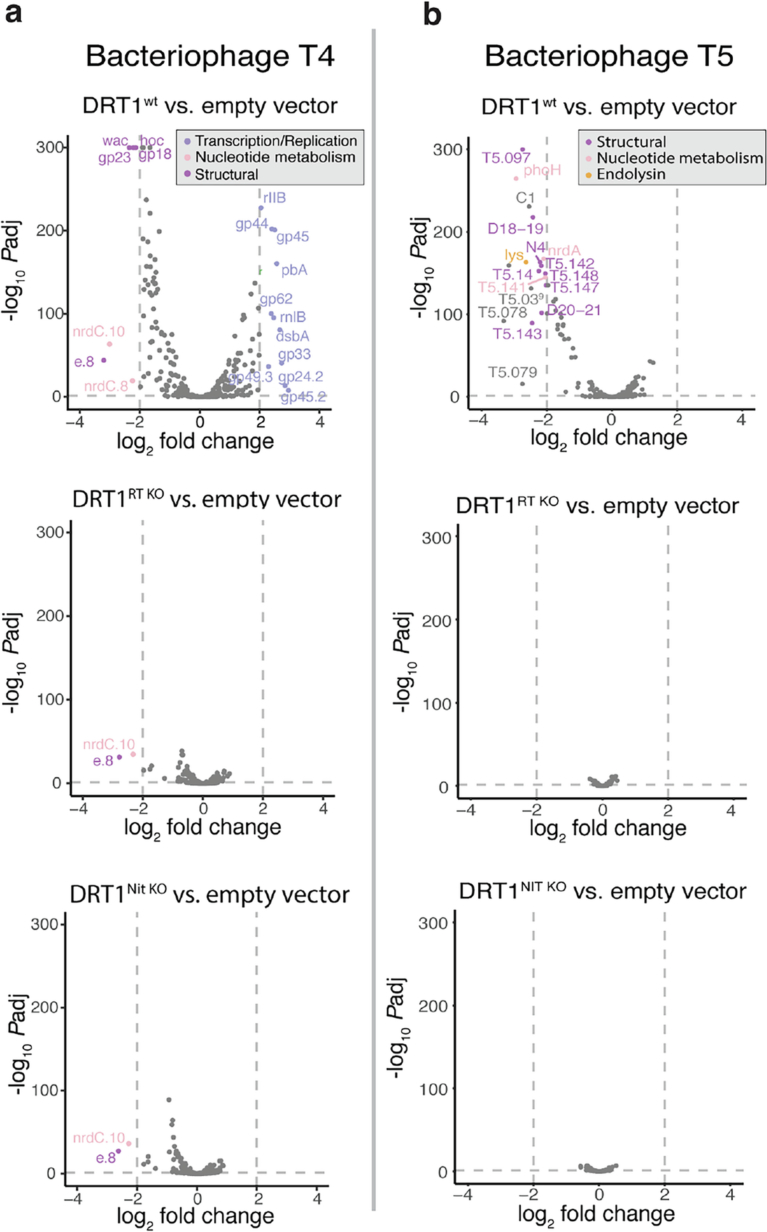
DRT1 alters bacteriophage transcription profiles. **a)** Volcano plot showing significantly upregulated and downregulated genes in the bacteriophage T4 genome upon exposure to DRT1^wt^, DRT1^RT KO^, DRT1^Nit KO^. Genes are colored according to function. Adjusted *P* values were calculated by DEseq2 using a two-sided Wald test followed by the Benjamini–Hochberg correction. **b)** Volcano plot showing significantly upregulated and downregulated genes in the bacteriophage T5 genome upon exposure to DRT1^wt^, DRT1^RT KO^, DRT1^Nit KO^. Genes are colored according to function. Adjusted *P* values were calculated by DEseq2 using a two-sided Wald test followed by the Benjamini–Hochberg correction. Source data

To understand bacteriophage gene association and response to DRT1, we performed RNA sequencing (RNA-seq) on T4- and T5-infected *E. coli* MG1655 expressing DRT1. Ten T4 mid-stage genes with known transcriptional or replication functions were significantly upregulated in DRT1-expressing strains, and five genes encoding structural proteins and nucleotide metabolism were downregulated (Extended Data Fig. 8a). Conversely, bacteriophage T5 only showed a significant downregulation of genes encoding structural proteins, nucleotide metabolism and endolysin (Extended Data Fig. 8b). Noteworthy changes to transcription profiles are the downregulated genes *nrdC.8* of T4 and *nrdA* of T5. Such nucleotide reductases have been shown to stimulate DRT9^17^. Both RT and nitrilase catalytic activity are required for significant alteration to relative bacteriophage transcriptome profiles (Extended Data Fig. 8). Together, these data suggest that DRT1 activation occurs during the mid-stage of bacteriophage infection.

**Fig. 5:**
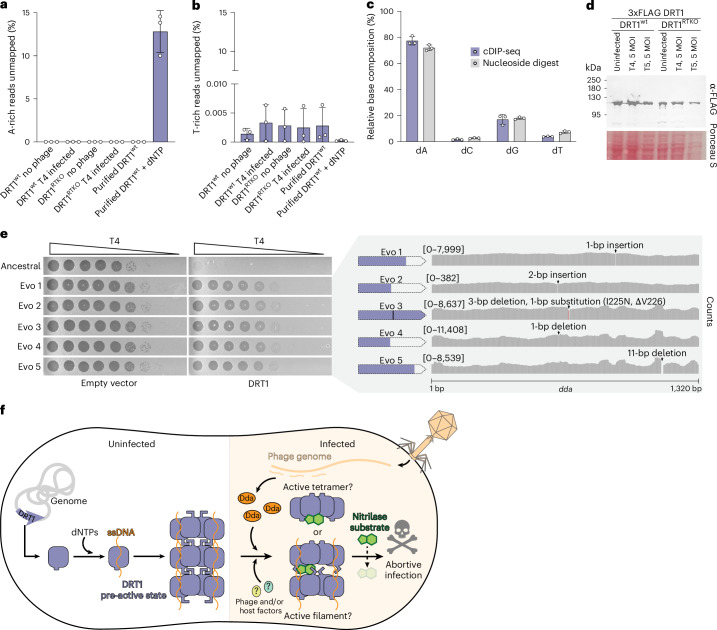
DNA adducts are a noncoding component of DRT1 defense. **a**, Percentage of unmapped reads containing greater than 70% adenine by cDIP-seq. Each sample shows *n* = 3 biological replicates, with data presented as mean and s.d. **b**, Percentage of unmapped reads containing more than 70% thymidine by cDIP-seq. Each sample shows *n* = 3 biological replicates, with data presented as mean and s.d. **c**, Comparison of nucleotide composition by cDIP-seq and LC nucleoside analysis of DNA synthesized by purified dNTP-reacted DRT1^wt^. Each sample shows *n* = 3 biological replicates, with data presented as mean and s.d. **d**, Western blot of DRT1^wt^ and DRT1^RTKO^ pre and post infection with bacteriophage. A representative blot of three biological replicates is shown. **e**, Evolution of bacteriophage T4 against DRT1^wt^, showing bacteriophage plaquing of five distinct evolutions (Evo) after the fifth round. Genomic sequencing results of the evolved bacteriophage are shown, with a zoomed-in view of the *dda* gene. **f**, Graphical model of the DRT1 mechanism. Source data

**Extended Data Fig. 9:**
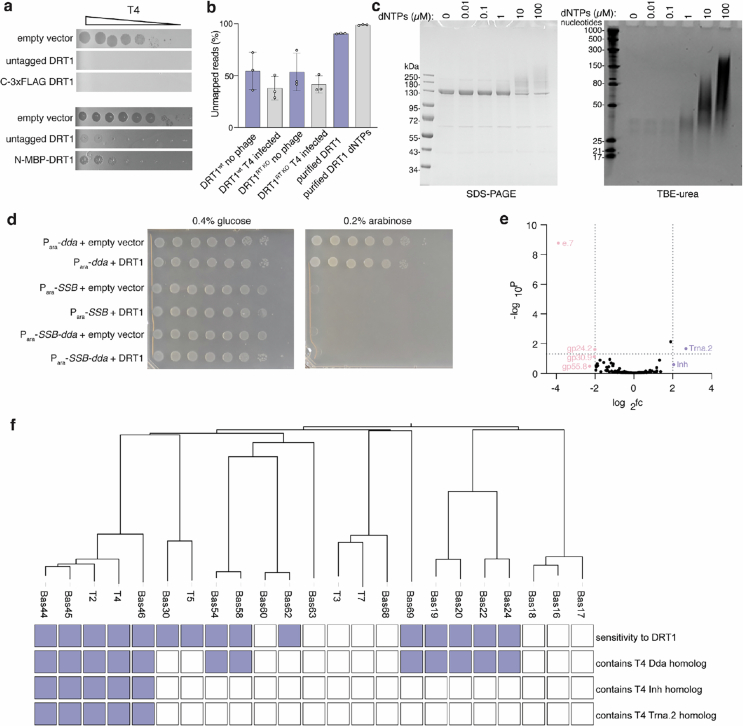
Viral determinants of DRT1 activation. **a)** Phage defense plaque assay of DRT1 with either no tag, a C-terminal 3xFLAG tag, or an N-terminal MBP tag. **b)** Comparison of total unmapped reads between cDIP-seq samples. **c)** Dose-response of DRT1^wt^ to dNTP mix titration. Samples run directly on SDS–PAGE (left) or digested with proteinase K and resolved on TBE-urea (right). **d)** Co-toxicity cell viability assay of DRT1^wt^ with candidate T4 phage trigger genes expressed from an arabinose-inducible promoter (P_ara_). **e)** LC-MS/MS proteomic analysis, volcano plot of differentially enriched T4 phage proteins following infection of cells expressing DRT1^wt^ versus DRT1^RT KO^. **f)** Phylogenetic tree of phage collection used in this study with indicators of sensitivity to DRT1, presence of Dda, Inh, Trna.2 or homologs. Source data

DRT9 produces long DNA adducts upon bacteriophage infection. Given that the mRNA levels of transcriptional and replication enzymes are upregulated during bacteriophage T4 infection, we postulated that DRT1 may function by a similar mechanism to DRT9. To determine whether phage infection alters the length or composition of the DNA adducts, we applied a variation of complementary DNA (cDNA) immunoprecipitation sequencing (cDIP-seq)^16,18^. First, we validated that adding either maltose-binding protein (MBP) or 3xFLAG tags to DRT1 did not affect antiphage defense (Extended Data Fig. 9a). Because nucleoside analysis indicated that DRT1 ssDNA is composed of at least 70% adenosine (A; Fig. 2f,g), we extracted and analyzed unmapped cDIP-seq reads containing ≥70% A (A-rich reads) obtained from DRT1 immunoprecipitation in the presence or absence of T4 bacteriophage infection. No increase in A-rich ssDNA or overall unmapped reads was observed in DRT1-expressing cultures upon T4 infection (Fig. 5a and Extended Data Fig. 9b). Indeed, only the positive control consisting of purified untagged DRT1 reacted in vitro with dNTPs showed a significant enrichment of A-rich reads, indicating that the ssDNA made by DRT1 in vivo is shorter than that made in vitro, regardless of bacteriophage infection. Consequently, cDNA complementary to the >70% adenosine-rich adducts (reads containing ≥70% thymidine) was not detected (Fig. 5b and Extended Data Fig. 9b). These data indicate that the A-rich ssDNA synthesized by DRT1 does not act as a template for DNA synthesis. Although no discernable sequence motif was identified in the ≥70% A reads, we observed that the base composition of purified DRT1 ssDNA determined via cDIP-seq matched closely to that determined for the same sample via liquid chromatography (LC) nucleoside analysis, supporting the validity of our ≥70% A filtering parameter (Fig. 5c). To test whether ssDNA length increases upon bacteriophage T4 infection, we conducted a western-blot analysis of lysate from DRT1-expressing cells after bacteriophage infection. DRT1 migration was not altered relative to RT-inactive DRT1 (Fig. 5d), consistent with the DRT1 ssDNA length remaining short in vivo, and that it is not appreciably extended upon bacteriophage infection. The migration of DRT1 by SDS–PAGE correlates with ssDNA length (Extended Data Fig. 9c). Together, these data support the function of DRT1 DNA adducts as structural elements rather than templates for toxic products.

Phages often develop resistance to defense systems by acquiring mutations in elements that activate the bacterial immune response. We serially passaged T4 and T5 phages on *E. coli* expressing DRT1 to isolate escape mutants. Bacteriophage T5 escapers were not observed, despite repeated attempts. However, five T4 mutants with resistance to DRT1 were isolated (Fig. 5e). Whole-genome sequencing revealed that all five escapers acquired unique insertions or deletions within the T4 *dda* 5′→3′ single-stranded DNA helicase gene, all of which would result in mutated amino acids or truncated protein (Fig. 5e). To test whether the Dda protein is sufficient to trigger DRT1, the wild-type (WT) *dda* sequence was cloned into an arabinose-inducible vector and co-expressed with DRT1 or empty vector. Coexpression of DRT1 and Dda was not toxic; that is, Dda is necessary, but not sufficient, to activate DRT1 (Extended Data Fig. 9d). Indeed, of the 15 bacteriophage sensitive to DRT1, 12 bacteriophage encode Dda or a Dda homolog (Extended Data Fig. 9f). All bacteriophage lacking Dda, or a homolog of Dda, are insensitive to DRT1. Given that the T4 single-stranded binding protein (SSB) is known to activate Dda^31^, we also tested the co-toxicity of SSB alone as well as dual expression with Dda. These data were inconclusive, as SSB was toxic even in the absence of DRT1 (Extended Data Fig. 9d). To further probe for binding of Dda or other bacteriophage proteins to DRT1, we conducted pulldown assays using tagged DRT1 followed by unbiased proteomics analysis. Only two T4 proteins—Inh and Trna.2—were significantly enriched in DRT1^wt^ relative to the DRT1^RTKO^ control (Extended Data Fig. 9e). Given that Inh and Trna.2 are only conserved in the four most closely related bacteriophage to T4 (Extended Data Fig. 9f) and that these proteins appear not to have core metabolic function, we did not attempt further characterization of these hits. Therefore, the complete activation pathway of DRT1 remains elusive.

## Discussion

There is substantial risk when generating a contextually toxic immunity protein. Safety mechanisms are essential to prevent unintentional programmed cell death. Here we determined that DRT1 performs template-free, protein-primed, DNA synthesis to simultaneously activate and inhibit a nitrilase effector domain. Covalent DNA adducts stabilize tetrameric DRT1, which further assembles into filaments. The tetrameric nitrilase assembly renders activity^32,33^, while the filamentous structure induces C-terminal domain-swapping to impose an inactive ‘closed-lid’ conformation over the apo-nitrilase active site^29^. Opening and closing of a similarly conserved inter-protomeric active-site lid is involved in the catalytic mechanism of other nitrilases^29,30^. At the mid-stage of infection, a 5′→3′ single-stranded DNA helicase along with other bacteriophage components activate DRT1 (Fig. 5f). Our data demonstrate that non-templated DNA synthesis acts as both an activator and safety mechanism for DRT1 and may also serve as a sensor of bacteriophage replication enzymes.

Nitrilase enzymes are typified by a supramolecular structure where homo-oligomeric assembly is a requirement for activity^32–35^. Consistent with this, RT-inactive DRT1—which is monomeric—is incapable of antiphage defense and is not toxic to the cell. Here we show that the DRT1 filament is inhibited by C-terminal domain-swapping due to confinement of conserved C-terminal residues that complete the nitrilase active site. We posit that filament rigidity locks the opening and closing of the C-terminal lid residues, thus preventing catalytic cycling until phage-triggered liberation. Mutating conserved C-terminal residues to disrupt domain-swapping abolishes antiphage defense, supporting their dual role in autoinhibition and catalysis. Indeed, the DRT1 C terminus mimics the closed conformation observed in other nitrilase structures. These observations raise the following question: what is the substrate of the DRT1 nitrilase? DRT1 activity results in abortive infection. As such, the nitrilase substrate is probably an essential molecule with amine or amide functional groups (for example, ATP, GTP, NAD^+^)^36–38^. Given the lack of precedent for nitrilases in antiphage defense, it is possible that DRT1 functions through an unorthodox pathway, generating a toxin upon activation.

It is reasonable to consider that DRT1, like DRT9, senses the nucleotide pool via DNA synthesis to enable selective filament formation^39,40^. Indeed, enzymes responsible for dNTP biosynthesis in phage T4 and T5 are downregulated in the presence of DRT1. However, unlike DRT9 and the abortive infection polymerases, protein-primed DNA synthesis is not sufficient for DRT1 to impart immunity. DNA adducts induce dormancy of DRT1 through nitrilase inhibition. Approximately 18% of *drt1* genes are adjacent to the transmembrane protein-encoding gene *drt1b*^6^. In such cases, *drt1b* may be essential for the activity of DRT1^5^, possibly functioning in a similar manner to the Hailong system where poly-dA adducts of HalB block the HalA ion channel. However, the majority of DRT1 operons lack *drt1b*, and, as shown here, DRT1 can impart robust immunity in the absence of DRT1b. We have uncovered no evidence to suggest that DRT1 DNA adducts perform any role beyond structural assembly, and perhaps sensing bacteriophage.

Most antiphage defense systems with supramolecular structure assemble catalytically active filaments^41–45^. The pre-formed assembly of quiescent DRT1 before phage infection parallels the mechanism of retrons^12–14^, where the effectors are inactivated by a DNA cage within filaments in the absence of infection. In this sense, DRT1 exemplifies a minimal retron composed of a single polypeptide chain capable of generating an msDNA analog to induce a pre-active state and potentially sense infection. It is enticing to speculate how Dda, and other phage components, may act upon DRT1-DNA adducts to induce an active state.

## Methods

Unless otherwise stated, all reagents are from New England Biolabs (NEB). DRT genes were synthesized and cloned into pACYC184, pBAD or pET by GenScript. The sequences of the encoded proteins are listed in Supplementary Table 1. All oligonucleotides were synthesized by Integrated DNA Technologies.

### DRT phylogenetic reconstruction

The RT dataset was obtained from the supplementary material of ref. ^6^. The alignment was processed using Geneious Prime, and sequences were exported as an aligned FASTA file. Within the alignment, a close homolog of DRT1^wt^—WP_075861988.1—which differs by only a single amino acid, was identified. This native sequence was replaced with DRT1^wt^ to maintain the original alignment. This modified dataset was used to construct the phylogenetic tree using FastTree^46^ with the LG^47^ evolutionary model and discrete gamma model with ten rate categories. To improve accuracy, four rounds of subtree-prune-regraft (SPR) moves and slow nearest-neighbor interchanges (NNI) were applied^46^. The tree was rooted at the midpoint and visualized using Interactive Tree of Life (iTol, https://academic.oup.com/nar/article/52/W1/W78/7645242).

### Phage defense plaque assays

All DRT systems were reconstituted in the surrogate host *E. coli* MG1655 from the low-copy plasmid pACYC184, with the DRT candidate gene replacing the tetracycline resistance gene normally encoded in pACYC184 while maintaining the original pTet promoter. *E. coli* MG1655 was made competent using the Mix & Go kit (Zymo Research, T3001), and successful transformants were selected on lysogeny broth (LB) agar containing 30 µg ml^−1^ chloramphenicol (LB-cam). Several colonies were grown in 9 ml of LB-cam overnight at 37 °C, and 200 µl of overnight culture was added to 6 ml of molten (51 °C) top agar (1% soy peptone, 0.5% NaCl, 0.05% MgCl_2_·6H_2_O, 10 mM MgSO_4_, 0.75% agar and 30 µg ml^−1^ chloramphenicol), mixed, and poured on 10-cm LB-cam agar plates. The plates were incubated at 37 °C for 5 h, dried for 5 min under laminar flow, then inoculated with phages. Tenfold serial dilutions of phage were prepared immediately before assay set-up in phage dilution (PD) buffer (50 mM Tris pH 7.5, 75 mM NaCl and 10 mM MgCl_2_). 3 µl of phage dilution was spotted, and the plates were incubated at 37 °C overnight.

### Liquid phage infection growth curve

Three different transformants of *E. coli* MG1655 harboring the indicated DRT plasmid were grown overnight in LB-cam. The cultures were diluted 1:100 in fresh LB-cam supplemented with 10 mM MgSO_4_ and grown until reaching an optical density at 600 nm (OD_600_) of ~0.3, then diluted to a final OD_600_ of 0.05 in the wells of a 96-well plate. Fresh dilutions of phage were prepared in LB-cam supplemented with 10 mM MgSO_4_ and added to each well to yield a final MOI of 0.1, 1 or 10. The final well volume was 150 µl. Growth kinetics were monitored at 37 °C with shaking in a BioTek Synergy H1 (Agilent) plate reader with OD_600_ reads every 5 min.

### Phage burst size assay

To determine the phage burst size and the efficiency of the center of infection, one-step growth curves were conducted as described in refs. ^15,48^, with slight adaptation. Overnight cultures of *E. coli* MG1655 harboring DRT1 in pACYC184 or empty vector were diluted 1:100 in LB-cam supplemented with 10 mM MgSO_4_, then grown to OD_600_ ≈ 0.6. A 2-ml sample of each culture was pelleted at 10,000*g* for 4 min, and resuspended in 900 µl of LB-cam supplemented with 10 mM MgSO_4_. T4 phage was added to MOI = 0.0002 and allowed to adsorb for 10 min (no shaking) at 37 °C. Unbound phage was removed by centrifuging at 10,000*g* for 2 min and resuspending in 300 µl of fresh medium, twice, then 10 µl of the suspension was added to 100 ml of pre-warmed LB-cam supplemented with 10 mM MgSO_4_ and grown at 37 °C with shaking at 200 r.p.m. One-milliliter aliquots were immediately withdrawn (15-min timepoint) and either directly plated for plaque enumeration (representing total phage in the sample) or vortexed with 50 µl of chloroform (representing free phage in the sample) then plated. Every 10 min thereafter, 1-ml aliquots were withdrawn and vortexed with 50 µl of chloroform before plaque enumeration. Plaque-forming units (p.f.u.) per ml per initial infection = (average titer of free phages at late timepoints)/initial infections, where initial infections = 15-min timepoint titer before chloroform treatment) − (15-min timepoint titer after chloroform treatment).

### Cell survival following phage infection

Overnight cultures of *E. coli* MG1655 harboring DRT1 in pACYC184 or empty vector were diluted 1:100 in LB-cam supplemented with 10 mM MgSO_4_, then grown to OD_600_ = 0.4. Aliquots of 2 ml of each culture were mixed with the indicated MOI of T4 phage and incubated with shaking at 37 °C for 15 min. Samples (1 ml) were withdrawn and centrifuged at 10,000*g* for 3 min. The supernatant was removed, and the pellet resuspended in 200 µl of LB. Resuspended cells were serially diluted in LB and plated on LB-cam plates. The plates were incubated at 37 °C overnight before enumerating the colonies.

### DRT1 expression and purification

All recombinant DRT proteins were expressed from the custom pET vector pMC009, which yields an N-terminal His14-MBP-SUMO fusion. Plasmids were transformed into T7 Express (NEB, C2566H) or One Shot BL21 Star (DE3) (Invitrogen, C601003). A colony from freshly transformed plates was used to inoculate 9 ml of LB (1% soy peptone, 0.5% yeast extract and 0.5% NaCl) supplemented with 50 µg ml^−1^ kanamycin (LB-kan) and grown overnight at 37 °C. This starter culture was diluted 100-fold into flasks containing 1 l of LB-kan, then grown at 37 °C until reaching an OD_600_ of ~0.6. Isopropyl-β-D-thiogalactoside (IPTG) was then added to 0.35 mM, and the cultures were incubated at 16 °C overnight before being harvested by centrifugation at 4,000*g* and 4 °C. The cell pellets were resuspended in lysis buffer (50 mM Tris pH 8.0, 500 mM NaCl, 5 mM MgCl_2_, 5 mM 2-mercaptoethanol, 2% glycerol and 0.1% Tween-20) supplemented with 1× Halt protease inhibitor cocktail (Thermo, 78438). The cells were lysed by sonication on ice for 3 min (10 s on, 10 s off) at 60% amplitude using a Q500 instrument (QSonica, Q500-110). The lysate was clarified by centrifugation, filtered (0.22 µm), then applied to three daisy-chained 5-ml MBPTrap HP columns (Cytiva, 28918779) equilibrated with lysis buffer. Protein was eluted in lysis buffer supplemented with 10 mM maltose. Fractions containing DRT were pooled and brought to 1 M NaCl final, followed by the addition of SENP1 SUMO protease (final concentration of 2 µM) and incubated overnight at 4 °C for tag cleavage. The sample was then loaded into a 3.5-kDa-cutoff bag (Spectrum Labs, 132726) and dialyzed against buffer containing 25 mM Tris pH 8.0, 1 M NaCl, 1 mM MgCl_2_, 5 mM 2-mercaptoethanol, 0.1% Tween-20 and 50% glycerol. Dialysis was used to concentrate, because we observed that DRT1 aggregates upon Centricon filtration. Concentrated samples were purified by SEC using a Superose 6 Increase 10/300 GL column (Cytiva, 29091596) equilibrated with SEC buffer (25 mM Tris pH 8.0, 250 mM NaCl, 1 mM MgCl_2_, 5 mM 2-mercaptoethanol and 2% glycerol). Fractions containing purified DRT protein were pooled, adjusted to 1 M NaCl final concentration, then mixed with 1 µM final SENP1 for a second round of tag cleavage overnight at 4 °C. Samples were concentrated by dialysis against buffer containing 25 mM Tris pH 8.0, 1 M NaCl, 1 mM MgCl_2_, 5 mM 2-mercaptoethanol and 50% glycerol using the same 3.5-kDa-cutoff membrane as above. Concentrated samples were loaded onto the Superose 6 Increase 10/300 GL column equilibrated with SEC buffer without glycerol (25 mM Tris pH 8.0, 250 mM NaCl, 1 mM MgCl_2_ and 5 mM 2-mercaptoethanol). Pure fractions were pooled and dialyzed against 25 mM Tris pH 8.0, 250 mM NaCl, 1 mM MgCl_2_, 5 mM 2-mercaptoethanol and 50% glycerol in 3.5-kDa-cutoff Slide-A-Lyzer cassettes (Thermo, 66333) to concentrate.

### DRT5S/L expression and purification

DRT5S/L was expressed as described above for DRT1. Cell pellets were resuspended in lysis buffer (50 mM Tris pH 8.0, 500 mM NaCl, 5 mM MgCl_2_, 5 mM 2-mercaptoethanol, 2% glycerol and 0.1% Tween-20) supplemented with 1× Halt protease inhibitor cocktail (Thermo, 78438). The cells were lysed by sonication on ice for 3 min (10 s on, 10 s off) at 60% amplitude using a Q500 instrument (QSonica, Q500-110). The lysate was clarified by centrifugation, 0.22-µm filtered, then applied to three daisy-chained 5-ml MBPTrap HP columns (Cytiva, 28918779) equilibrated with lysis buffer. Protein was eluted in lysis buffer supplemented with 10 mM maltose. The N-His-MBP tag was not cleaved.

### Intact DRT1 LC-MS analysis

LC-MS was conducted using a Vanquish Flex UHPLC system coupled to an Orbitrap Eclipse mass spectrometer (Thermo Scientific). WT and dRT (100 fmol µl^−1^) were reduced with 20 mM tris(2-carboxyethyl)phosphine (TCEP; Thermo Scientific) for 10 min at 37 °C in starting mobile phase conditions. Two picomoles (20 μl) was injected for separation on a 2.1 × 50-mm PLRP-S analytical column (5 μm, 1,000 Å, Agilent Technologies), using the following mobile phases: A, 0.1% difluoroacetic acid (DFA) in water; B, 0.1% DFA in acetonitrile; and a 10-min gradient from 20–68% B at 0.2 ml min^−1^. Mass spectra were acquired in low-pressure ‘intact protein’ mode using a HESI source, ion funnel RF 80%, source fragmentation 20 V, source voltage 3.8 kV, source and vaporizer temperatures of 300 °C, sheath gas 35 (a.u.) and aux gas 10 (a.u.). Mass spectra were collected at 7.5k resolution, AGC 1E5, with 50-ms max injection time. UniDec deconvolution software^49,50^ was used for intact mass determinations.

### Peptide LC-MS/MS using product-ion-triggered EThcD

DRT1 (20 µg) was digested according to the S-Trap micro spin column protocol (Protifi). Proteolytic digestion using 2 µg of trypsin (New England Biolabs) was carried out at 37 °C for 18 h. Tryptic peptides were recovered by sequential elution with 0.2% formic acid in water, 50% acetonitrile and 70% acetonitrile. The combined eluate was evaporated and reconstituted in 0.1% formic acid and 2% acetonitrile in water. After *A*_205_ quantification using a Nanodrop One spectrophotometer (Thermo Scientific), the peptide concentration was adjusted to 200 ng µl^−1^ for LC-MS/MS analysis.

Peptides were separated on an EASY-nLC 1200 system using an EASY-Spray C-18 analytical column (150 mm, 75 µm, 3 µm, 100 Å) coupled to an Orbitrap Eclipse mass spectrometer (Thermo Fisher Scientific), then 200 ng of peptides were separated using the following mobile phases: A, 0.1% formic acid in water; B, 0.1% formic acid in 80% acetonitrile; and a 75-min linear gradient from 2% to 45% B at 300 nl min^−1^. MS1 spectra were collected at 60k resolution with a 1.5-s cycle time. MS/MS spectra were collected by data-dependent acquisition using HCD (25 NCE) with ion trap detection (1E5 AGC; *m*/*z* 110–2,000), and a 10-s dynamic exclusion after two events (±10-ppm tolerance). An extended mass range of *m*/*z* 110–4,000 was used for product-ion-triggered EThcD, 30k orbitrap resolution using calibrated charge-dependent ETD reaction times, 5E5 AGC and 25% supplemental HCD.

### Characterization of nucleotide-modified peptides

Byonic (Protein Metrics) was used to identify peptides and site-localize nucleotide-modified amino acids using diagnostic fragment ions^51^ (Supplementary Table 2). Spectra from an initial untargeted LC-MS/MS run were evaluated using Byonic MS/MS filtering to determine whether any contained the diagnostic fragments listed in Supplementary Table 2. If at least two diagnostic fragments for a given nucleotide were observed in the top ten most abundant ions, the product-ion-triggered EThcD LC-MS/MS method was implemented in a second analysis. After filtering by peptide spectral match quality (Byonic Score and PEP 2D score), the paired product-ion-triggered HCD/EThcD MS/MS spectra were manually evaluated to confirm modifications at a specific amino-acid residue.

### Mass photometry

The samples were analyzed with a Refeyn TwoMP mass photometer on cation-coated slides (Refeyn, MP-CON-71002). Uncoated slides (Refeyn, MP-CON-41001) yielded similar results. The mass standard curve was constructed using the MassFerence P1 calibrant (Refeyn, MP-CON-41033) diluted into SEC buffer. The reaction mixtures contained 50 mM Tris pH 7.5, 150 mM NaCl, 5 mM MgCl_2_, 2 mM dithiothreitol (DTT), ~0.5 mg ml^−1^ DRT1 protein and 0.1 mM dNTP mixture or individual ddNTP where indicated. This mixture was incubated for 15 min at 37 °C, then diluted into SEC buffer to yield DRT1 at a final coverslip conentration of ~500 nM.

### dNTP polymerization assays

Template-independent DNA polymerization was assayed in reactions containing 50 mM Tris pH 7.5, 150 mM NaCl, 5 mM MgCl_2_, 2 mM DTT, 0.4 mg ml^−1^ RNAse A (NEB, T3018L) and ~0.5 mg ml^−1^ DRT protein. This mixture was incubated for 15 min at 37 °C to digest any contaminating RNA that might be present. DNA synthesis was then initiated by adding 0.1 mM of each dATP, dCTP, dGTP and dTTP, unless otherwise indicated. Reactions were incubated at 37 °C for 30 min. For SDS–PAGE analysis, the reactions were quenched by mixing with SDS sample dye (NEB, B7703S) and heating at 95 °C for 5 min, followed by electrophoresis on 4–20% Novex gel (Invitrogen, XP04200BOX) and staining with Coomassie blue.

For nucleic acid analysis, the reactions were quenched by adding 25 mM EDTA and 0.4 mg ml^−1^ proteinase K (NEB, P8107S), followed by incubation for an additional 15 min at 37 °C to release DNA covalently attached to protein. To visualize products via electrophoresis, the reactions were mixed 1:1 with RNA loading dye (NEB, B0363S), heated at 65 °C for 5 min, then resolved on 15% tris/borate/EDTA (TBE)-urea gels (Invitrogen, EC6885BOX). The gels were stained for 15 min with 1× SYBR Gold (Invitrogen, S11494) dissolved in 1× TBE buffer, then rinsed briefly with distilled water and imaged under UV light. Low-range ssRNA ladder (NEB, N0364S), in some cases mixed with microRNA marker (NEB, N2102S), was used to approximate the size of the ssDNA products, given the absence of a commercial ssDNA ladder in this size range.

### Nucleoside analysis liquid chromatography

DRT reactions (100 µl) were prepared as described in the ‘dNTP polymerization assays’ section. Nucleic acid was released by digestion with 0.4 mg ml^−1^ proteinase K for 15 min at 37 °C. The resulting sample was mixed with 700 µl of binding buffer and purified using the NEB Monarch spin PCR and DNA cleanup kit (T1130S). Purified DNA was eluted in water and then digested into nucleosides with NEB nucleoside digestion mix (M0649S). For quantitative comparison of the relative nucleoside composition, the raw integral corresponding to each nucleoside peak was divided by its respective extinction coefficient at 260 nm: A, 15,200; C, 7,050; G, 12,010; T, 8,400 (ref. ^52^).

### Negative-stain electron microscopy

Unreacted DRT1 samples or DRT1 reacted with dNTPs were diluted to 0.3 mg ml^−1^ in buffer containing 50 mM Tris pH 7.5, 150 mM NaCl and 5 mM MgCl_2_. The diluted samples were immediately applied to glow-discharged copper-supported carbon 400 mesh grids (Electron Microscopy Sciences) and stained with 2% methylamine tungstate (Nano-W, Nanoprobes). The grids were loaded onto either a JEOL NEOARM (200 kV) or JEOL 1400F (120 kV) transmission electron microscope, each equipped with a OneView camera. Micrographs were acquired in 4k × 4k mode using Digital Micrograph (Gatan) with a nominal magnification of ×50,000 (pixel size = 2.7 Å) on the NEOARM and ×60,000 (pixel size = 3 Å) on the 1400F. Micrographs were exported to cryoSPARC^53^ for contrast transfer function (CTF) correction, particle picking and 2D classification. 3D volumes were generated using ab initio reconstruction, and the data were processed through heterogeneous and homogeneous refinements. Structural figures were produced using ChimeraX v1.8^54^.

### Cryo-EM sample preparation and data collection

Samples containing 0.6 mg ml^−1^ DRT1 + dNTPs were applied to C-flat 1.2/1.3 300 mesh grids (Electron Microscopy Sciences) that had been glow-discharged using a Leica EM ACE600 instrument for 30 s with 20-mA current. Using a Leica EM GP2 automatic plunging system set to 12 °C and 100% humidity, 4 μl of sample was applied to the backside of the grid and excess liquid was blotted away by contact with filter paper for 3 s. Sample application and blotting were repeated twice (three blots total) before plunge-freezing the grid in liquid ethane.

A total of 10,188 movies were collected from a single grid using a Titan Krios TEM (Thermo Fisher) equipped with a K3 direct electron detector, Volta phase plate and Gatan imaging filter with slit width of 20 eV. All movies were collected using SerialEM automation software^55^. Particles were imaged at a calibrated magnification of 0.413 Å pixel^−1^, with an exposure of ~18 eps for 2.8 s for a total exposure of ~50 e Å^−2^. Details of the data collection parameters are provided in Table 1.

### Cryo-EM data processing and structure building

Motion correction and CTF estimation were performed using cryoSPARC v4.6.0 live processing^53^. After all data were collected, pre-processed exposures were exported, and further processing was performed using cryoSPARC. Initial 2D classification was performed using 31 particles that were manually picked along the filamentous particle and extracted. The two resulting classes were used to guide additional particle picking using the filament tracer job with the filament diameter set to 140 Å, a separation distance of 0.85 diameters, and a minimum filament length of two diameters considered. The picks were then extracted and distributed into two classes containing particles. 176,480 particles were used to generate an ab initio reconstruction of three classes. The full particle stack containing 921,286 particles was carried into a heterogeneous refinement of the three classes, and the particles from the highest-quality class were used for homogeneous refinement of the best volume with no applied symmetry. Next, a helical refinement job was performed with no applied symmetry or helical parameters defined. The resulting volume was then analyzed using a symmetry search job set to search over a helical rise of 40–140 Å and helical twist of −180°–180°. The search identified a helical order of 2, rise of 59.13 Å and twist of −121.22°. The 3D volume was then subjected to another round of helical refinement using these parameters and *D*2 point group symmetry. Local CTF refinement was performed on the refined volume and particle stack, and a final helical refinement was performed using non-uniform refinement with minimization over per-particle scale factors (input values, not reset to 1.0), including the defined helical parameters and applied *D*2 symmetry. An initial model of the complex was generated using AlphaFold 2 (https://alphafoldserver.com). The highest-confidence output model was docked into the refined volume via ChimeraX v1.8^54^. The structure was iteratively refined and completed using a combination of Phenix v1.21.2, Coot v0.9.2 and ISOLDE v1.8^56–58^.

### Exonuclease protection assay

DRT1 was first reacted with dNTPs to yield extended ssDNA adducts. Reactions (20 µl) containing 0.5 mg ml^−1^ DRT1, 50 mM Tris pH 7.5, 150 mM NaCl, 5 mM MgCl_2_, 2 mM DTT, 0.4 mg ml^−1^ RNase A (NEB, T3018L) and 0.1 mM final concentration dNTP mix (NEB, N0447L) or an equivalent volume of water were reacted for 15 min at 37 °C, then 2.2 µl of ExoI reaction buffer and 1 µl ExoI (NEB, M0293L) or water were added to each reaction and incubated for 30 min at 37 °C. The reactions were heated to 93 °C for 10 min to inactivate the ExoI, then mixed with 1 µl of proteinase K (NEB, P8107S) and incubated for 30 min at 37 °C. The samples were mixed with an equal volume of RNA loading dye, heated at 65 °C for 5 min, then resolved on 15% TBE-urea gel followed by SYBR Gold staining.

### Illumina sequencing of DRT1 pulldown ssDNA

The protocol for sequencing of ssDNA produced by DRT1 during phage infection was adapted from the cDIP-seq technique of ref. ^16^. *E. coli* MG1655 was transformed with empty plasmid or plasmid encoding N-MBP-tagged DRT1 WT or its D324N, D325N mutant. Colonies were inoculated into LB-cam, grown overnight at 37 °C, then subcultured into 200 ml of fresh LB-cam supplemented with 10 mM MgSO_4_ and grown to an OD_600_ of 0.8. T4 phage, or an equivalent volume of phage buffer, was then added to yield an MOI of 5 or 0. Cells were incubated with phage for 10 min at 37 °C with 200-r.p.m. shaking, then pelleted by centrifugation for 10 min at 10,000*g* at 4 °C. The pellet was flash-frozen on dry ice, then stored at −80 °C.

The pellets were resuspended in 30 ml of pulldown lysis buffer (50 mM Tris pH 8.0, 250 mM NaCl, 5 mM MgCl_2_, 2% glycerol, 0.1% Tween-20, 2 mM DTT, 1× Halt inhibitor) and sonicated at 25% amplitude for 1 min total (2 s on, 10 s off). Cell debris was pelleted by centrifugation at 20,000*g* for 20 min at 4 °C. Supernatants were mixed with 6 ml of 50% slurry amylose resin (NEB, E8022L) that had been pre-washed three times in pulldown lysis buffer. The lysates were rocked in 50-ml conical tubes with resin for 1 h at 4 °C. The resin was gently pelleted by centrifugation for 1 min at 500*g*, the supernatant was discarded, then the pellet was resuspended in 30 ml of fresh pulldown lysis buffer. This was repeated twice more for a total of three washes. Protein was eluted by adding 1 ml of pulldown lysis buffer without Tween-20 and supplemented with 50 mM maltose, and rocked for 30 min at 4 °C. The total protein in the eluates was quantified with a detergent-compatible Bradford assay (Thermo, 23246). The samples were normalized based on total protein, and 50-µl aliquots each containing ~20 µg of total protein were used for downstream purification.

Aliquots of eluate were mixed with 3 µl of RNase A (NEB, T3018L) and incubated at 37 °C for 15 min. Proteinase K (NEB, P8107S) was then added (3 µl) and the samples digested for 1 h at 37 °C. The samples were purified using an ssDNA/RNA clean and concentrate kit (Zymo, D7011). Purified ssDNA was quantified via Qubit (Thermo, Q10212). Control samples consisting of 20 µg of fully purified untagged DRT1 with or without in vitro reaction with 0.5 mM dNTPs were also subjected to the above steps.

Approximately 5 ng of ssDNA from each sample was used as starting material for the xGen ssDNA and Low-Input DNA Library Prep Kit (IDT 10009859). Library preparation followed the manufacturer’s instructions, with the following changes to SPRISelect bead volumes to maximize the capture of short (<200 nt) ssDNA. For post-extension cleanup, 1.8× SPRISelect bead volume was used and only a single bead purification step was done. For post-ligation cleanup, 1.4× SPRISelect bead volume was used. Indexing polymerase chain reaction (PCR) used 12 cycles with xGen UDI primers (IDT 10005975). Post-PCR cleanup used a 1× SPRISelect bead volume. Samples were sequenced on an Illumina Miseq set-up with 75 cycle single-end reads.

Trim Galore was used to remove adapter sequences and remove reads shorter than 10 bp. Reads were mapped to a reference file containing the MG1655 genome (NC_000913.3), T4 genome (GenBank: AF158101.6) and relevant plasmid sequences, using bwa with default settings. SAMtools (v 1.21) flagstat was used to analyze alignment statistics and count unmapped reads. SAMtools fasta was used to extract unmapped reads. A custom Python script was used to filter unmapped reads based on polyA or polyT content, with reads containing ≥70% A or ≥70% T being extracted for downstream analysis.

### Evolution of escape mutant phage

Escape mutant phages were evolved as described in ref. ^59^. For both phages T4 and T5, five independent phage populations were evolved against resistant host *E. coli* MG1655 pACYC-DRT1 in 96-well deep-well plates. In each plate, a control population was evolved with only the sensitive host containing empty pACYC plasmid. Overnight cultures in LB-cam were diluted 1:100, grown to an OD_600_ of ~1.0, then diluted again to an OD_600_ of 0.00125 in either Teknova LB (L8000) supplemented with 1 mM MgSO_4_ and 30 µg ml^−1^ chloramphenicol for T4, or Teknova Minimal M9 broth (M8000) supplemented with 30 µg ml^−1^ chloramphenicol for T5. Each well of the plate was seeded with 200 µl of diluted bacteria. Each well was infected with 20 µl of tenfold serial dilutions of T4 or T5 in PD buffer, or 20 µl of PD buffer only to monitor contamination. The plates were sealed with breathable film and incubated at 37 °C with 300 r.p.m. shaking for 18 h. The plate was harvested by pooling the most diluted well that was fully cleared along with the first uncleared well for each population. The pooled samples were centrifuged at 4,000*g* for 20 min at 4 °C to pellet cell debris. The supernatant was passed through 0.22-µm filters (Millipore UFC30GV00) and stored in tubes, with 40 µl of chloroform added to prevent bacterial contamination.

### Evolved phage DNA extraction and Illumina sequencing

To prepare for DNA extraction of evolved phages, each DRT1-evolution population and each corresponding empty-vector control population were plated on a soft agar overlay of DRT1-expression or empty-vector bacteria, respectively. A single plaque from each sample was used to infect 10-ml cultures of the same bacterial host grown to an OD_600_ of 0.5 in LB-cam supplemented with 10 mM MgSO_4_. Cultures were grown for 5 h at 37 °C to allow phage propagation. The cultures were centrifuged at 4,000*g* for 20 min at 4 °C, and supernatant was decanted into fresh tubes containing 1 g of PEG 8000 (10% final) and 0.58 g NaCl (1 M final). The tubes were mixed by inversion until dissolved, then incubated at 4 °C overnight. They were then centrifuged at 4,000*g* for 30 min to pellet phages, which were resuspended in 500 µl of 5 mM MgSO_4_. Each sample received 1.25 µl of DNase1 (NEB, M0303S) and 1.25 µl of RNAse A (NEB, T3018L) followed by incubation at 37 °C for 1 h. Each sample then received 30 µl of 0.5 M EDTA pH 8.0 (30 mM final), 25 µl of 10% SDS (0.5% final) and 1.25 µl of proteinase K (NEB, P8107S) (20 µg total), followed by 1 h of incubation at 60 °C. The samples were cooled to room temperature then mixed with an equal volume of phenol:chloroform (1:1). Following centrifugation for 10 min at 10,000*g*, the supernatant was transferred to fresh tubes and phenol:chloroform extraction was repeated. An equal volume of chloroform was mixed with the supernatant and centrifuged for 10 min at 10,000*g*. Supernatant was transferred to fresh tubes and mixed with a 1:10 volume of 3 M sodium acetate pH 5.2 and 2.5 volumes of ice-cold 100% ethanol. The mixture was incubated on ice for 30 min to precipitate DNA, which was then pelleted by centrifugation for 20 min at 16,000*g* and 4 °C. The DNA pellet was washed twice with 500 µl of 70% ethanol, then dried with tube caps open for 15 min. The DNA was resuspended in 0.1× TE buffer (1 mM Tris-HCl pH 8.0, 0.1 mM EDTA).

Purified phage DNA was diluted to 50 ng µl^−1^ and sheared using a Covaris ML230 instrument in 8-AFA tubes (Covaris, 520292) to yield DNA fragments of ~175 bp. Sheared DNA was then prepared for sequencing using an NEBNext Ultra II library prep kit for Illumina (NEB, E7645S) and NEBNext multiplex oligos (NEB, E6446S). Agilent Tapestation was used to verify library quality and concentrations. Libraries were sequenced using Illumina NextSeq500 with 150-bp paired-end reads.

Reads were aligned to the reference T4 genome (GenBank: AF158101.6) using BWA and Samtools. The consensus assembly of each sample was then extracted using Samtools. The extracted assembly of the DRT1 evolution clones was then aligned to the extracted assembly of the empty plasmid control evolution in Geneious Prime 2024-10-19. Coverage tracks were visualized in IGV.

### Bacteriophage phylogenetic tree

Viral phylogenetic analysis was carried out by the VICTOR web service (https://ggdc.dsmz.de/victor.php)^60^. Pairwise comparisons of the amino-acid sequences were conducted using the Genome-BLAST Distance Phylogeny (GBDP) method^61^ under settings recommended for prokaryotic viruses^60^. The resulting intergenomic distances were used to infer a balanced minimum evolution tree with branch support via FASTME including SPR postprocessing^62^ for using the D6 distance formula. The tree was rooted at the midpoint and visualized using iTOL (https://academic.oup.com/nar/article/52/W1/W78/7645242).

### Homolog identification

Structures were predicted for all proteins annotated in the 23 phage genomes, using AlphaFold2^20^ through ColabFold^63^. Predicted structures for T4 Dda (UniProt: P32270), Inh (UniProt: P18058) and Trna.2 (UniProt: P13324) were used as queries in a Reseek^64^ search, in ‘sensitive’ mode, against a database of the phage predicted structures. Hits with a Reseek Alignment Quality (AQ) score above 0.75 were considered homologs.

### Whole-transcriptome sequencing

*E. coli* MG1655 transformed with DRT constructs in pACYC184 were grown overnight in LB-cam at 37 °C. The bacteria were diluted 100-fold into fresh LB-cam supplemented with 10 mM final MgSO_4_ and grown at 37 °C to an OD_600_ of ~0.25. T4 phage was then added at 1 MOI, calculated assuming 8 × 10^8^ bacterial cells in 1 ml of culture at an OD_600_ of 1.0. T5 phage was added at 5 MOIs. Samples (1 ml) were dispensed into the wells of a 24-well plate. The plate was incubated at 37 °C for 30 min with 240-r.p.m. shaking, then centrifuged for 10 min at 3,500*g* and 4 °C to pellet the cells. The supernatant was discarded and the pellets frozen on dry ice. Thawed pellets were resuspended in 25 µl of TBS (50 mM Tris pH 7.0, 150 mM NaCl) and 10 µl of T4 lysozyme (NEB, P8115L) and incubated for 5 min at 25 °C. The RNA was then purified according to the manufacturer’s specifications for enzymatic lysis using the Monarch Total RNA Miniprep Kit (NEB, T2010S), beginning with the addition of 300 µl of RNA lysis buffer to each sample after lysozyme. The RNA integrity number was verified to be above 7 for all samples using an Agilent Tapestation. 500 ng of total RNA per sample was then depleted of ribosomal RNA using the NEBNext rRNA depletion kit (Bacteria) (NEB, E7850L). cDNA libraries were produced using the NEBNext Ultra II Directional RNA Library Prep Kit for Illumina (NEB, E7760S), with PCR amplification via single-index NEBNext Multiplex Oligos (NEB, E7335S and E7730S). Library quality and concentrations were verified with Agilent Tapestation. Libraries were sequenced via Illumina NextSeq500 using 75-bp paired-end reads.

Transcriptomic data were processed using the following tools in the Galaxy server and R. Adapters were trimmed with Trim Galore using default parameters, followed by quality control using FastQC. Reads were mapped to either the T4 phage genome (GenBank: AF158101.6) or T5 phage genome (NCBI: NC_005859.1) using HISAT2 with strandedness set to reverse (RF). Mapped reads were counted using htseq-count with strandedness set to reverse. Differential gene expression was determined using DESeq2, with independent filtering and outlier filtering turned off.

### Western blotting

Overnight cultures of *E. coli* MG1655 transformed with pACYC184 encoding C-terminal 3xFLAG-tagged DRT1 were diluted 100-fold into fresh LB-cam supplemented with 10 mM final MgSO_4_ and grown at 37 °C to an OD_600_ of ~0.3, at which point 5 MOIs of phage (or an equal volume of phage dilution buffer) were added. The cultures were then incubated at 37 °C for 20 min and collected by centrifugation at 4,000*g* for 10 min. The pellets were resuspended in 500 µl of IP lysis buffer (20 mM Tris-HCl, pH 7.5, 150 mM KCl, 2 mM MgCl_2_, 0.2% Triton X-100, 1× Halt protease inhibitor), then lysed via sonication (20% amplitude for 1 min 30 s, 2 s on 5 s off). Debris was cleared by centrifugation at 10,000*g* for 5 min. The supernatant was mixed with SDS loading dye, heated at 95 °C for 5 min, then run on 4–20% gel (Novex XP04205BOX) at 200 V for 1 h. Proteins were transferred to PVDF membrane (Biorad, 1620260) using the BioRad Turbo Transfer system. The membranes were washed 3× with distilled water, stained with Ponceau, briefly destained in distilled water, then dried on filter paper at room temperature for 1 h. The membranes were blocked by rocking with 5% milk in Tris-buffered saline with Tween-20 (TBST) at 4 °C overnight. Primary mouse anti-FLAG antibody (Sigma, F1804) was added to the blocking solution at 1:1,000 and rocked for 2 h at 4 °C. The membranes were washed 3× in TBST, then incubated with DyLight 800 goat anti-mouse 4×PEG conjugate secondary antibody (Invitrogen, SA5-35521) in TBST at 1:100,000 for 1 h at room temperature. After washing 3× in TBST, the membranes were imaged with a Licor Odyssey instrument.

### Pulldown and mass spectrometry

Protein samples for pulldown analysis were prepared as described in the ‘Illumina sequencing of DRT1 pulldown ssDNA’ section. Biological triplicates of each sample type were reduced, alkylated, digested and chromatographically separated using the methods outlined in the ‘Peptide LC-MS/MS using product-ion-triggered EThcD’ section.

For this analysis, an Orbitrap Exploris mass spectrometer (Thermo Fisher Scientific) was used, equipped with a FAIMS Pro interface (Thermo Fisher Scientific). A 160-min linear gradient was used from 2% to 35% B at 300 nl min^−1^. The FAIMS Pro module was used to further separate peptides by cycling the compensation voltage (CV) between −50 and −70 V. For each CV, MS1 spectra spanning *m*/*z* 400–1,450 were collected at 120k resolution. MS/MS spectra were collected by data-dependent acquisition using HCD (30 NCE, 45k resolution) with a 1.5-s cycle time (2E5 AGC), a 45-s dynamic exclusion after a single event (±10-ppm tolerance) and an automatic *m*/*z* scan range with a 1.2 *m*/*z* isolation window. Raw data were searched using two reference proteomes and a purified protein FASTA: the *E. coli* K-12 strain MG1655 proteome, T4 phage proteome and MBP-DRT1 sequences. Raw LC-MS/MS data were analyzed using the LQF-MBR workflow within FragPipe (FragPipe v22.0; MSFragger v4.1; IonQuant v1.10.27; Philosopher v5.11; Python 3.11.11). The results were processed using Fragpipe Analyst v1.18 using an intensity-based DDA LFQ approach^65^ and an all-pairs approach to compare each experimental condition with Perseus-type imputation and Benjamini–Hochberg false discovery rate (FDR) correction^66^.

### Coexpression toxicity assays

The toxicity of co-expressing DRT1 with candidate phage trigger proteins was assayed as described in ref. ^67^. Briefly, single colonies of *E. coli* MG1655 harboring pACYC184-DRT1 or empty pACYC184 plus pBAD encoding the indicated phage protein were grown for 6 h at 37 °C in LB–glucose to saturation. A 200-µl aliquot of each culture was pelleted at 4,000*g* for 10 min, washed once in 1× PBS, and resuspended in 400 μl of 1× PBS. The cells were then serially diluted tenfold in 1× PBS, and 3 µl was spotted on plates composed of M9 medium (6.4 g l^−1^ Na_2_HPO_4_.7H_2_O, 1.5 g l^−1^ KH_2_PO_4_, 0.25 g l^−1^ NaCl, 0.5 g l^−1^ NH_4_Cl) supplemented with 0.1% casamino acids, 0.4% glycerol, 2 mM MgSO_4_, 0.1 mM CaCl_2_, 5% Teknova LB (vol/vol) and 1.5% agar (wt/vol). The plates were incubated overnight at 37 °C.

### Reporting Summary

Further information on research design is available in the Nature Portfolio Reporting Summary linked to this Article.

## Online content

Any methods, additional references, Nature Portfolio reporting summaries, source data, extended data, supplementary information, acknowledgements, peer review information; details of author contributions and competing interests; and statements of data and code availability are available at 10.1038/s41594-026-01813-8.

## Supplementary information

Supplementary Information Supplementary Table 1 Sequences used in this study. Supplementary Table 2 Nucleotide ion masses used to identify modified peptides.

Reporting Summary

## Source data

Source Data Fig. 1 Numerical source data for Fig. 1b.

Source Data Fig. 2 Numerical source data for Fig. 2f,g.

Source Data Fig. 3 Unprocessed gel for Fig. 3e.

Source Data Fig. 4 Numerical source data for Fig. 4f.

Source Data Fig. 5 Numerical source data for Fig. 5a,b,c.

Source Data Fig. 5 Unprocessed western blot for Fig. 5d.

Source Data Extended Data Fig. 1 Unprocessed gels for Extended Data Fig. 1d,e,f.

Source Data Extended Data Fig. 1 Numerical source data for Extended Data Fig. 1a,b,c.

Source Data Extended Data Fig. 2 Unprocessed gel for Extended Data Fig. 2b.

Source Data Extended Data Fig. 2 Numerical source data for Extended Data Fig. 2a.

Source Data Extended Data Fig. 5 Unprocessed gel for Extended Data Fig. 5d.

Source Data Extended Data Fig. 5 Numerical source data for Extended Data Fig. 5e.

Source Data Extended Data Fig. 8 Numerical source data for Extended Data Fig. 8a,b.

Source Data Extended Data Fig. 9 Unprocessed gel for Extended Data Fig. 9c

Source Data Extended Data Fig. 9 Numerical source data for Extended Data Fig. 9b,e.

## Data Availability

Atomic coordinates and electron density maps have been deposited to the Protein Data Bank. The accession number for the DRT1 atomic model is PDB ID 9YFD and for the density map is EMD-72883. All nucleic acid sequencing data have been deposited to the National Center for Biotechnology Information BioProject PRJNA1348522. Proteomics data can be accessed at https://repository.jpostdb.org/entry/JPST004145.0. Source data are provided with this paper.

## Code availability

A Python script for analyzing nucleoside composition from pulldown sequencing is freely available on GitHub (https://github.com/Chang-Hwa/DRT_paper).
