# Supplementary figures and images for: Semirandom DNA adducts regulate a filamentous defense-associated reverse transcriptase
Source: Nat Struct Mol Biol. 2026 Jun 10;33(6):953–61. doi: 10.1038/s41594-026-01813-8 (PMC13275509; doi:10.1038/s41594-026-01813-8)

**Fig.3e. Uncropped gel.**

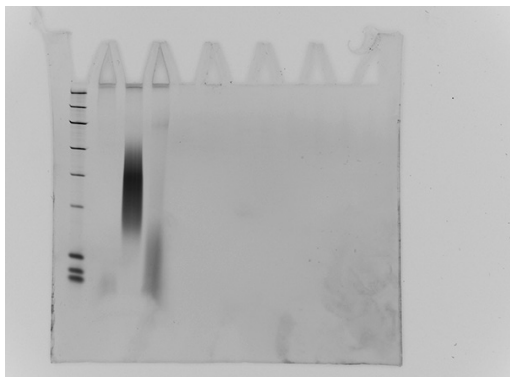

Supplement: Supplementary file 5 — Unprocessed gel for Fig. 3e. [file 41594_2026_1813_MOESM5_ESM.pdf]

**Fig.5d. Uncropped western blot and ponceau S-stained membrane.**

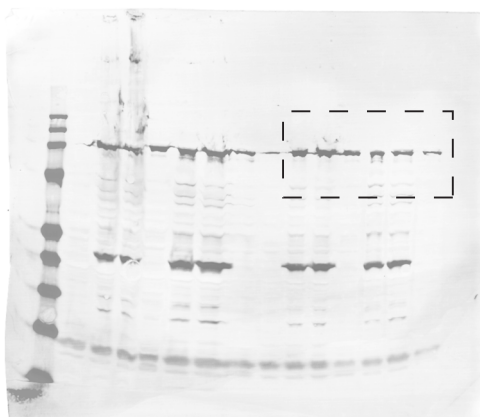

anti-FLAG

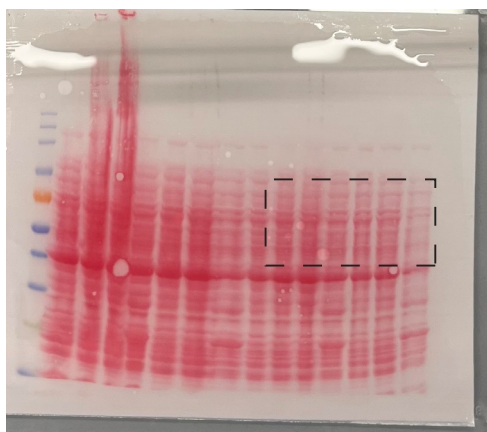

Ponceau S

Supplement: Supplementary file 8 — Unprocessed western blot for Fig. 5d. [file 41594_2026_1813_MOESM8_ESM.pdf]

Extended Data Fig. 1. Uncropped gels.

Fig. 1.d

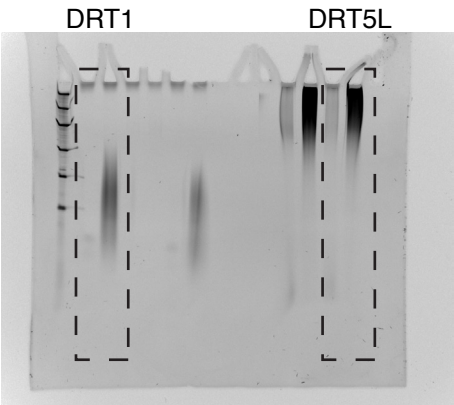

Fig. 1.d

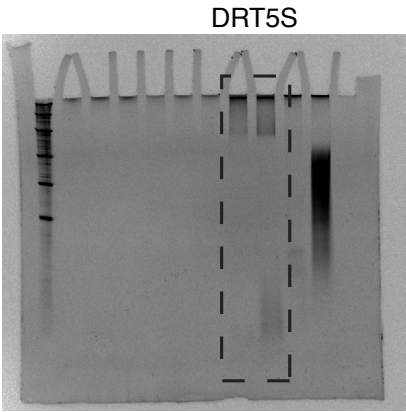

Fig. 1.e

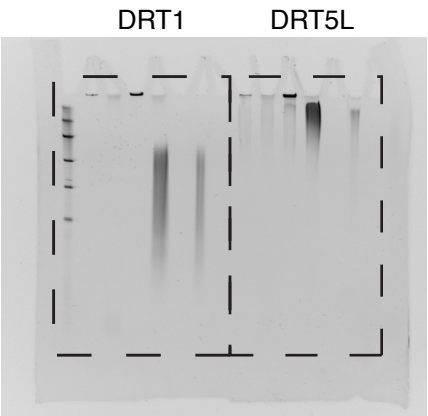

Fig. 1.f

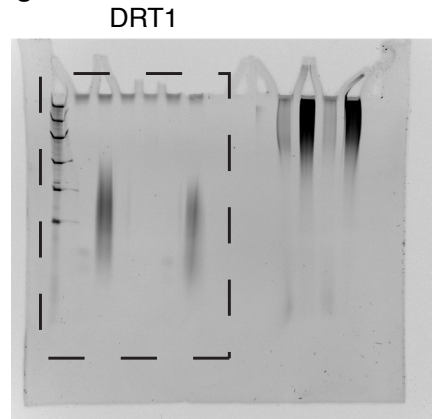

Supplement: Supplementary file 9 — Unprocessed gels for Extended Data Fig. 1d,e,f. [file 41594_2026_1813_MOESM9_ESM.pdf]

Extended Data Fig. 2b. Uncropped gel

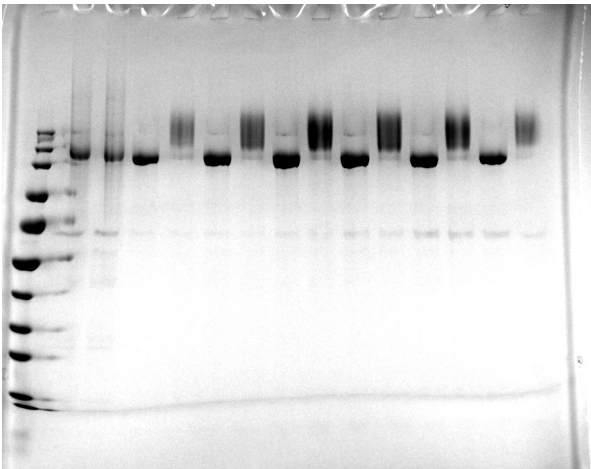

Supplement: Supplementary file 11 — Unprocessed gel for Extended Data Fig. 2b. [file 41594_2026_1813_MOESM11_ESM.pdf]

**Extended Data Fig. 5d. Uncropped gel**

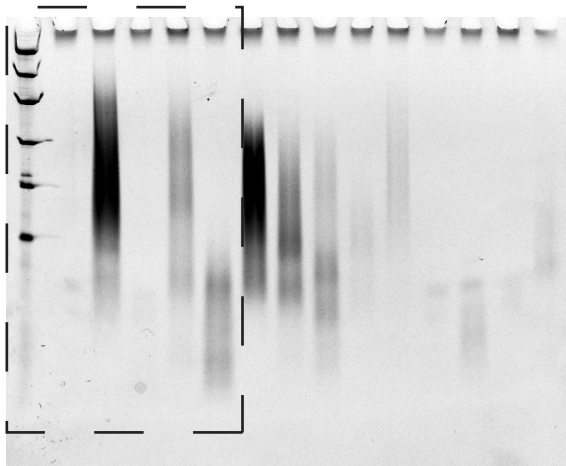

Supplement: Supplementary file 13 — Unprocessed gel for Extended Data Fig. 5d. [file 41594_2026_1813_MOESM13_ESM.pdf]

Extended Data Fig. 9c. Uncropped gels

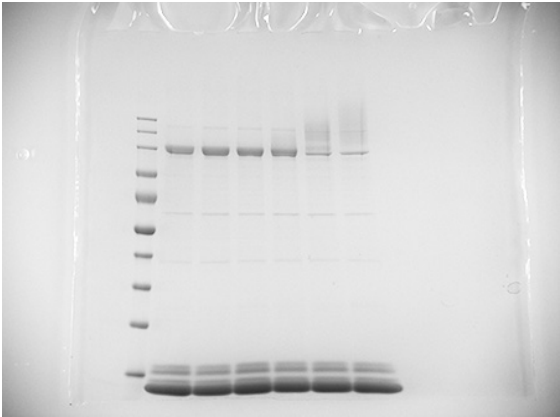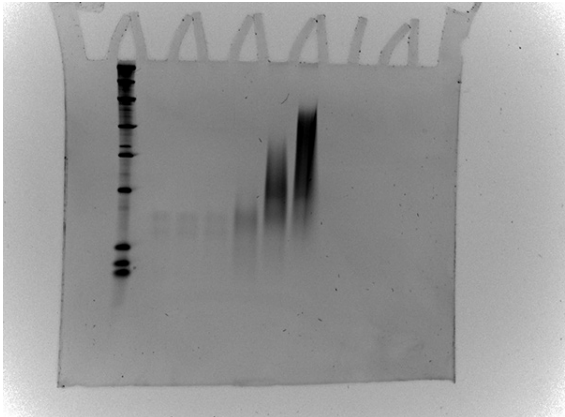

Supplement: Supplementary file 16 — Unprocessed gel for Extended Data Fig. 9c [file 41594_2026_1813_MOESM16_ESM.pdf]
